# Identification of complex *Plasmodium falciparum* genetic backgrounds circulating in Africa: a multicountry genomic epidemiology analysis

**DOI:** 10.1016/j.lanmic.2024.07.004

**Published:** 2024-12

**Authors:** Olivo Miotto, Alfred Amambua-Ngwa, Lucas N Amenga-Etego, Muzamil M Abdel Hamid, Ishag Adam, Enoch Aninagyei, Tobias Apinjoh, Gordon A Awandare, Philip Bejon, Gwladys I Bertin, Marielle Bouyou-Akotet, Antoine Claessens, David J Conway, Umberto D'Alessandro, Mahamadou Diakite, Abdoulaye Djimdé, Arjen M Dondorp, Patrick Duffy, Rick M Fairhurst, Caterina I Fanello, Anita Ghansah, Deus S Ishengoma, Mara Lawniczak, Oumou Maïga-Ascofaré, Sarah Auburn, Anna Rosanas-Urgell, Varanya Wasakul, Nina F D White, Alexandria Harrott, Jacob Almagro-Garcia, Richard D Pearson, Sonia Goncalves, Cristina Ariani, Zbynek Bozdech, William L Hamilton, Victoria Simpson, Dominic P Kwiatkowski

**Affiliations:** a Mahidol–Oxford Tropical Medicine Research Unit, Faculty of Tropical Medicine, Mahidol University, Bangkok, Thailand; b Centre for Tropical Medicine and Global Health, University of Oxford, Oxford, UK; c Medical Research Council Unit The Gambia at London School of Hygiene & Tropical Medicine, Banjul, The Gambia; d London School of Hygiene and Tropical Medicine, London, UK; e West African Centre for Cell Biology of Infectious Pathogens, University of Ghana, Accra, Ghana; f Institute of Endemic Diseases, University of Khartoum, Khartoum, Sudan; g Department of Obstetrics and Gynecology, College of Medicine, Qassim University, Buraydah, Saudi Arabia; h Department of Biomedical Sciences, School of Basic and Biomedical Sciences, University of Health and Allied Science, Ho, Ghana; i Department of Biochemistry and Molecular Biology, University of Buea, Buea, Cameroon; j KEMRI Wellcome Trust Research Programme, Kilifi, Kenya; k Institute of Research for Development, Paris, France; l Faculty of Medicine, University of Health Sciences, Libreville, Gabon; m LPHI, MIVEGEC, INSERM, CNRS, IRD, University of Montpellier, Montpellier, France; n Malaria Research and Training Centre, University of Science, Techniques and Technologies of Bamako, Bamako, Mali; o National Institute of Allergy and Infectious Diseases, National Institutes of Health, Rockville, MD, USA; p Noguchi Memorial Institute for Medical Research, Accra, Ghana; q National Institute for Medical Research, Dar Es Salaam, Tanzania; r Department of Biochemistry, Kampala International University in Tanzania, Dar es Salaam, Tanzania; s Wellcome Sanger Institute, Hinxton, UK; t Bernhard Nocht Institute for Tropical Medicine, Hamburg, Germany; u Menzies School of Health Research, Charles Darwin University, Darwin, NT, Australia; w Institute of Tropical Medicine Antwerp, Antwerp, Belgium; x School of Biological Sciences, Nanyang Technological University, Singapore; y MRC Centre for Genomics and Global Health, Big Data Institute, Oxford University, Oxford, UK

## Abstract

**Background:**

The population structure of the malaria parasite *Plasmodium falciparum* can reveal underlying adaptive evolutionary processes. Selective pressures to maintain complex genetic backgrounds can encourage inbreeding, producing distinct parasite clusters identifiable by population structure analyses.

**Methods:**

We analysed population structure in 3783 *P falciparum* genomes from 21 countries across Africa, provided by the MalariaGEN Pf7 dataset. We used Principal Coordinate Analysis to cluster parasites, identity by descent (IBD) methods to identify genomic regions shared by cluster members, and linkage analyses to establish their co-inheritance patterns. Structural variants were reconstructed by de novo assembly and verified by long-read sequencing.

**Findings:**

We identified a strongly differentiated cluster of parasites, named AF1, comprising 47 (1·2%) of 3783 samples analysed, distributed over 13 countries across Africa, at locations over 7000 km apart. Members of this cluster share a complex genetic background, consisting of up to 23 loci harbouring many highly differentiated variants, rarely observed outside the cluster. IBD analyses revealed common ancestry at these loci, irrespective of sampling location. Outside the shared loci, however, AF1 members appear to outbreed with sympatric parasites. The AF1 differentiated variants comprise structural variations, including a gene conversion involving the *dblmsp* and *dblmsp2* genes, and numerous single nucleotide polymorphisms. Several of the genes harbouring these mutations are functionally related, often involved in interactions with red blood cells including invasion, egress, and erythrocyte antigen export.

**Interpretation:**

We propose that AF1 parasites have adapted to some unidentified evolutionary niche, probably involving interactions with host erythrocytes. This adaptation involves a complex compendium of interacting variants that are rarely observed in Africa, which remains mostly intact despite recombination events. The term cryptotype was used to describe a common background interspersed with genomic regions of local origin.

**Funding:**

Bill & Melinda Gates Foundation.

## Introduction

The protozoan *Plasmodium falciparum*, a leading cause of malaria, is responsible for hundreds of thousands of deaths yearly in sub-Saharan Africa.^1^ This parasite has shown great propensity for genetic changes in response to human interventions, often undermining malaria control and elimination efforts.^2^ The availability of high-throughput genome sequencing has made it possible to study such changes in near-real time, providing important insights into the dynamics of evolution at the population level.^3^, ^4^, ^5^ In particular, studies of *P falciparum* population structure–the differences in the distribution of genetic variation between populations–have revealed insights into *P falciparum* demography by identifying patterns associated with deviations from random mating.

**Research in context**

**Evidence before this study**

This study builds on previous work by the authors to elucidate regional population structure, particularly identifying sub-populations driven by artemisinin resistance in the Greater Mekong subregion, and resistance to drugs in Africa and Oceania. Here, we sought to identify new population structure patterns in Africa, applying methods based on identity by descent (IBD) algorithms. We searched PubMed, without language or start date restrictions, up to Jan 30, 2024, for relevant literature (terms: falciparum, ("population structure" OR subpopulations), "identity by descent") yielding nine peer-reviewed publications, including four studies that analysed data from the MalariaGEN whole-genome sequence dataset. Although most studies were on a national scale, we reviewed one global study, along with regional studies from the Greater Mekong subregion, South America, and Africa. Regional studies from Africa describe results complementary to those presented here, showing a divergence of the Ethiopian *P falciparum* parasite population.

**Added value of this study**

We analysed population structure by clustering *P falciparum* genomes by similarity and by extent of IBD. Due to high transmission and frequent recombination, African parasites are mostly expected to exhibit low levels of similarity. However, we found a group of parasites (named AF1), present at low frequency across the continent, whose members share several portions of the genome. The genomic regions forming this complex genetic background appear to be co-inherited and in strong linkage disequilibrium. These regions are also strongly differentiated, comprising many loci (>20) that carry alleles rarely seen in other African parasites, including large structural variants. Despite this constellation of co-inherited loci, AF1 parasites show evidence of recombination with local non-AF1 individuals, such that some degree of geographical differentiation is seen within the group. The most shared loci within AF1 contain genes known to interact with host erythrocytes, participating in invasion and egress, or exporting antigens to the red blood cell surface.

**Implications of all the available evidence**

This study has identified a novel phenomenon in malaria genetic epidemiology, which we dubbed cryptotype, because we the identification of AF1 required specific analyses of ancestry. Although previous studies have found subpopulations of highly similar parasites, these were typically localised geographically and driven by recent selection. The geographical extent of the AF1 population, from Madagascar to Mauritania, indicates it is neither localised nor recent. Its discovery suggests we need to rethink our understanding of *P falciparum* epidemiology and evolution. How is such a complex constellation of mutated loci maintained, despite the extremely low likelihood of passing it on intact to the progeny after recombination? One possible explanation is that AF1 occupies a niche in which the ensemble of mutations provides an adaptation that confers a survival advantage. The functions of the genes involved suggest that this involves host–parasite interactions, but further studies will be required to elucidate the underlying biology. Meanwhile, the present work provides experimental parasitologists with a catalogue of candidate interacting variants that can form the basis for new investigations.

Where malaria transmission is high, large parasite populations and frequent infection rates provide frequent mating opportunities for genetically distinct parasites, maintaining high levels of genetic variation through outbreeding. Hence, genetic distances within these populations tend to be evenly distributed, without marked population structure, as seen in parts of Africa.^6^ In areas of low malaria transmission, however, mosquitoes often acquire parasites from a single infected individual, which results in mating between clones with identical genomes (ie, selfing). High levels of selfing result in inbred populations, which exhibit lower genetic distances between individuals, and can be detected in population genomics analyses. High levels of inbreeding can also occur when selfing is beneficial for parasite survival. Specifically, when mating with a wild-type parasite, a single beneficial mutation will propagate to only half of the offspring. By contrast, selfing allows the mutation to be passed onto all offspring. Population structure driven by drug-resistant mutations was observed in southeast Asia, where inbred artemisinin-resistant populations were associated to mutations in the *kelch13* gene.^7^^,^^8^ The benefits of high selfing rates are even greater when transmitting complex genetic backgrounds, for example, when a drug-resistant mutation is detrimental to parasite development unless accompanied by multiple compensatory mutations.^9^ In the case of artemisinin resistance in the Greater Mekong subregion, at least five loci were found to be co-inherited with key *kelch13* mutations.^8^ The greater the number of co-inherited loci in a genetic background, the more recombination reduces the likelihood that a complete set of variants will be passed on to offspring during outbreeding. However, if the full set of variants strongly increases survival likelihood, then lineages from selfing parasites could undergo selection, resulting in reduced genetic variation.

Analyses of population structure in sub-Saharan Africa have shown high levels of genetic variations in high-transmission regions, with gradual genetic differentiation between east and west Africa.^10^ Population structure can be observed at the margins of endemicity, in lower transmission regions such as The Gambia and the Horn of Africa.^10^^,^^11^ To date, however, no published analyses have reported population structure driven by the selection of complex co-inherited multilocus genetic backgrounds.

We conducted an analysis of African genomes from the MalariaGEN Pf7 dataset^6^ to search for patterns of population structure associated with complex genetic backgrounds. By applying methods based on identity by descent (IBD), we characterised a group of parasites, labelled AF1, which share a complex multilocus genetic background, suggesting that its components are co-inherited. AF1 parasites are found at low frequency across Africa, from Mauritania to Madagascar. We defined the term cryptotype to describe their genetic background, reflecting the fact that it is hidden by large portions of the genome that bear similarities to other local parasites. We investigated functional relationships between the cryptotype component loci, and the forces that could be contributing to the maintenance of this complex and geographically widespread genetic background.

## Methods

The process of selection of samples and variants is detailed in the appendix (p 2), but we provide a summary. We began with the MalariaGEN Pf7 dataset,^6^ which comprises 20 864 samples. We selected samples with very low within-sample diversity (within-sample *F* statistic [*F*_*WS*_]≥0·95) from Africa, discarding those that had high genotyping missingness, resulting in a set of 3783 samples, organised by macroregions: west Africa, central Africa, and east Africa (table). Samples were genotyped at 743 584 high-quality biallelic single nucleotide polymorphisms (SNPs) that had a minor allele frequency (MAF) of at least 0·1% in at least one macroregion. Samples were genotyped at each SNP with the allele supported by the most reads. Allele frequencies were estimated at each SNP by calculating the proportion of samples carrying each allele, disregarding samples with missing genotypes. Fixation indices (*F*_*ST*_) between each pair of populations were estimated at each SNP as previously described.^8^ The AF1 mean *F*_*ST*_ was calculated as the arithmetic mean of *F*_*ST*_ between AF1 and each of the macroregions (west Africa, central Africa, and east Africa). *F*_*ST*_ estimation was also performed at 68 360 additional SNPs that had high levels of missingness in samples processed with selective whole-genome amplification (sWGA; appendix p 2).^12^

**Table:** Summary of sample counts by country in each macroregion

	Country code	Sample count	AF1 Count	Percentage of AF1	95% CI	p value
**West Africa**						
Mauritania	MR	49	1	2·0%	0–12·0	0·46
Mali	ML	534	4	0·7%	0·2–2·0	0·40
Senegal	SN	110	0	0	0–4·1	0·65
The Gambia	GM	462	3	0·6%	0·1–2·0	0·27
Guinea	GN	70	5	7·1%	2·7–16·0	0·0012
Ghana	GH	1191	19	1·6%	1·0–2·5	0·21
Côte d’Ivoire	CI	43	1	2·3%	0–13·0	0·42
Burkina Faso	BF	11	0	0	0–30·0	1·00
Benin	BJ	88	0	0	0–5·0	0·63
Nigeria	NG	52	0	0	0–8·2	1·00
Cameroon	CM	127	0	0	0–3·5	0·41
**Central Africa**						
Gabon	GA	33	1	3·0%	0–17	0·34
DR Congo	CD	186	1	0·5%	0–3·3	0·73
**East Africa**						
Sudan	SD	24	0	0	0–16·0	1·00
Ethiopia	ET	19	0	0	0–20·0	1·00
Kenya	KE	356	1	0·3%	0–1·7	0·12
Uganda	UG	5	1	20·0%	2·0–4·0	0·061
Tanzania	TZ	290	4	1·4%	0·4–3·6	0·78
Malawi	MW	100	5	5·0%	1·9–11·0	0·0044
Mozambique	MZ	15	0	0	0–24·0	1·00
Madagascar	MG	18	1	5·6%	0–28·0	0·20
**Total**		3783	47	1·2%	0·8–1·4	

Each row represents one African country where *Plasmodium falciparum* samples analysed in this study were sampled. Countries are grouped by macroregion in which the country is located (west, central, or east Africa). The columns show the name of the country and its ISO 3166 code; the total number of analysed samples from that country; the number of AF1 samples identified in the country, their percentage of the samples analysed (with 95% CI); and the p value of a Fisher’s exact test comparing the proportion within the country against the proportion in the rest of the continent. p values less than 0·01 represent high statistical significance. ISO=International Organization for Standardization.

Genotype analyses were performed using bespoke software programs written in Java (Java Development Kit version 17) and R (version 4.4.0). Principal coordinate analysis (PCoA) was conducted using cmdscale in the R stats package with a NxN pairwise genetic distance matrix (N=3783). PCoA is a method that maps samples onto a series of dimensions (principal components) to explain variance in a genetic distance matrix, clustering together highly similar genomes. Genetic distances were estimated by the proportion of the 743 584 SNPs in which two samples carry different alleles, after discarding SNPs for which one or both samples have a missing genotype. AF1 proportions and 95% CIs were calculated by R DescTools package (version 0.99.5419) using the Agresti–Coull method. The linkage disequilibrium measure *r*^*2*^ was computed for all pairs of SNPs with mean *F*_*ST*_ of at least 0·2 (appendix p 2). Circular genome linkage disequilibrium plots were generated using Circos (version 0.69).^12^

IBD analysis was performed using the program hmmIBD^13^ with default parameters. We filtered out extremely low-frequency variants, retaining coding SNPs with MAF of at least 0·1 in at least one macroregion, and at least one sample with a non-reference genotype. High-IBD regions were defined by identifying uninterrupted sequences of SNPs in which at least 50% of all AF1 pairs were in IBD; neighbouring high-IBD regions separated by gaps of 50 kbp or less were subsequently merged.

De novo assemblies of genomic sequencing reads were performed using Cortex version 1.0.5.21^14^ with k-mer size of 61. The generated contigs were aligned against reference sequences provided by the Pf3k project using BioEdit (version 7.2.5). Sequencing reads coverage visualisations were produced using the LookSeq web application^15^ and JBrowse2 (version 2.10.3).^16^ *MSP1* gene references were obtained from GenBank, accession numbers X03371.1 (K1), AB276005.1 (RO33), and X05624.2 (MAD30). Functional information about genes was obtained from PlasmoDB and literature searches.

### Role of the funding source

The funder had no role in study design, data collection, data analysis, data interpretation, or writing of the report.

## Results

### Population structure analysis of African *P falciparum* parasites

We selected 3783 samples from the quality filtered MalariaGEN Pf7 analysis dataset,^6^ which were essentially clonal (*F*_*WS*_ ≥0·95), and had low genotype missingness (table). We estimated allele frequencies in three macroregions: west Africa, central Africa, and east Africa for all high-quality biallelic SNPs in Pf7, and discarded SNPs with a MAF of less than 0·1% in all three macroregions, yielding a set of 743 584 SNPs to be used in our analyses.

**Figure 1:**
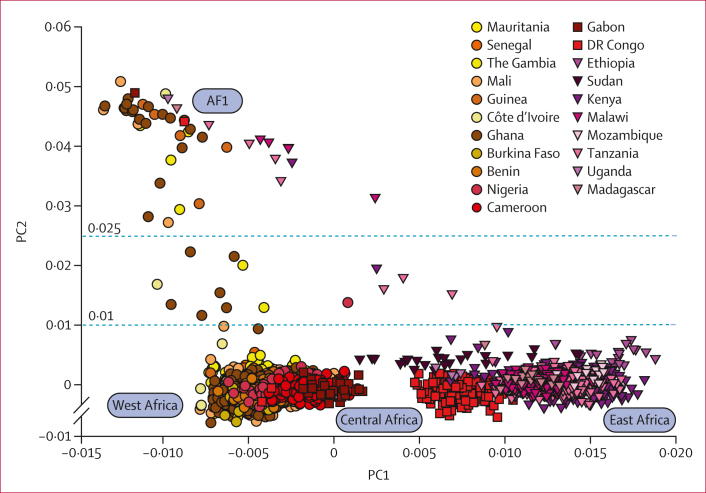
PCoA of African samples, revealing population structure A plot of PC2 versus PC1 is shown. Along PC1 (explaining 1·9% of variance), samples separate geographically so that the east Africa, central Africa, and west Africa macroregions can be distinguished as labelled. A cluster of AF1 parasites, originating from multiple countries, separates along PC2 (0·9% of variance). Two horizontal dotted lines indicate the thresholds for defining the AF1 population. Samples with PC2 of more than 0·025 were classified as AF1; those with PC2 of less than 0·01 were classified as non-AF1; the remaining parasites were disregarded in further analysis, because their AF1 membership status is inconclusive. PCoA=principal coordinate analysis. PC1=first principal component. PC2=second principal component.

PCoA plots showed that the first component (PC1) was driven by the differentiation between parasites from west Africa and east Africa (figure 1), as reported previously.^10^ Unexpectedly, the second component (PC2) was driven by a diverging cluster, which we named AF1, composed of parasites from multiple countries across Africa, rather than from sites in close geographical proximity. The broad geographical distribution of AF1, including regions of high transmission, suggests that population structure is not driven by low endemicity. The broad geographical distribution of AF1, including regions of high transmission, suggests that population structure is not driven by inbreeding due to low endemicity. Instead, the observed population structure is more likely to be caused by portions of the genome where AF1 members share a high degree of similarity, which differentiates them from other individuals within the same countries.

**Figure 2:**
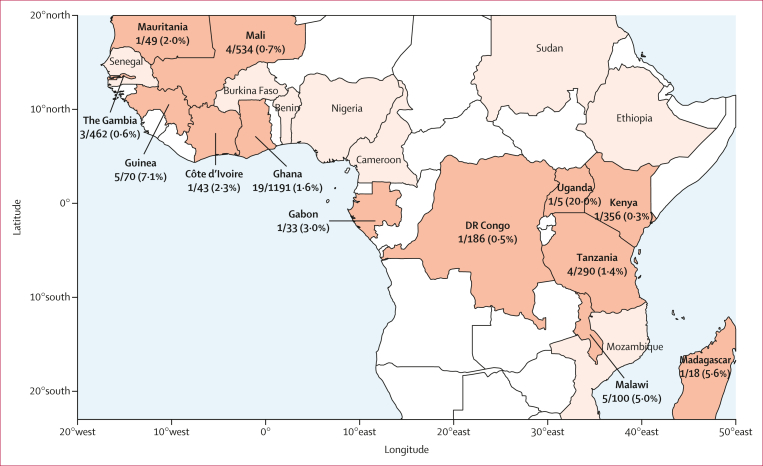
Geographical distribution of AF1 parasite samples In the map shown, countries from which parasites were sampled are shown with a coloured background and a label showing the country name. Countries where AF1 parasites were found are shown with an orange background. For each of the countries where AF1 parasites were found, the number of AF1 samples and the total number of analysed samples are separated by a solidus, and the percentage of AF1 samples is shown in brackets. The map uses data from Natural Earth.

We labelled samples with a PC2 of at least 0·025 as AF1 members (figure 1), whereas 3722 (98·4%) of 3783 parasites had a PC2 of 0·01 or less and were labelled according to their macroregion (west Africa, central Africa, or east Africa). Samples with PC2 values between 0·01 and 0·025 (14 [0·37%] of 3783 total samples) were disregarded. AF1 members comprised 47 (1·2%) of 3783 total samples in the set, sampled from 13 countries across all macroregions, up to 7500 km apart (figure 2). Within most countries, AF1 accounts for 1–6% of samples, with significantly higher proportions in Guinea and Malawi only (table). AF1 frequencies were also consistent by year, except for a higher proportion in 2011 (appendix p 6), which is difficult to interpret because it coincided with the collections of samples in Guinea and Malawi. To a first level of approximation, AF1 appears to be evenly distributed at low frequency across the continent.

### Genetic features of AF1

The clustering of AF1 parasites suggests they share alleles that are uncommon in other African populations. To identify differentiated sites, we estimated allele frequencies in AF1, west Africa, central Africa, and east Africa at all coding SNPs, to calculate the mean *F*_*ST*_ between AF1 and each of the other populations. For this task, we included 68 360 additional SNPs that had low coverage in sWGA samples (appendix p 2). This analysis revealed 198 coding non-synonymous SNPs with mean *F*_*ST*_ of 0·5 or more, 71 (36%) of which had mean *F*_*ST*_ of 0·75 or more (appendix p 7). The differentiated SNPs are not evenly distributed across the genome, but clustered in several regions on multiple chromosomes (appendix p 17). We found high-*F*_*ST*_ variant clusters in chromosomes 1, 2, 4, 9, 10, 11, 13, and 14, whereas other chromosomes showed lower differentiation levels. The clustering of high-*F*_*ST*_ SNPs suggests that AF1 characteristic loci contain highly differentiated long haplotypes. Although most SNP clusters occupy regions of less than 100 kbp, one locus on chromosome 10 stretches over approximately 250 kbp, possibly indicating a large structural variant.

**Figure 3:**
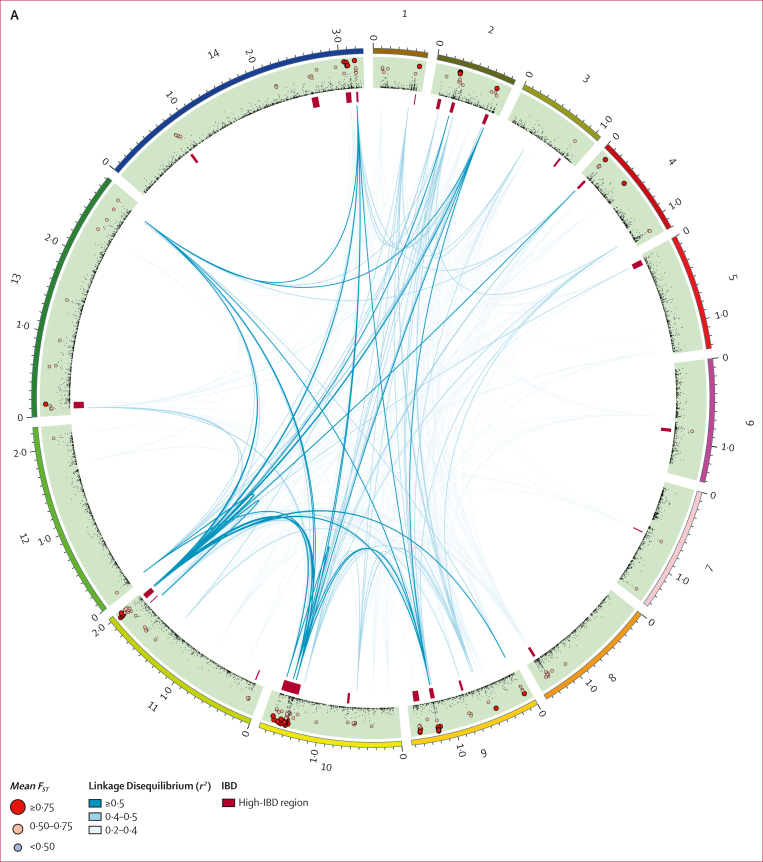

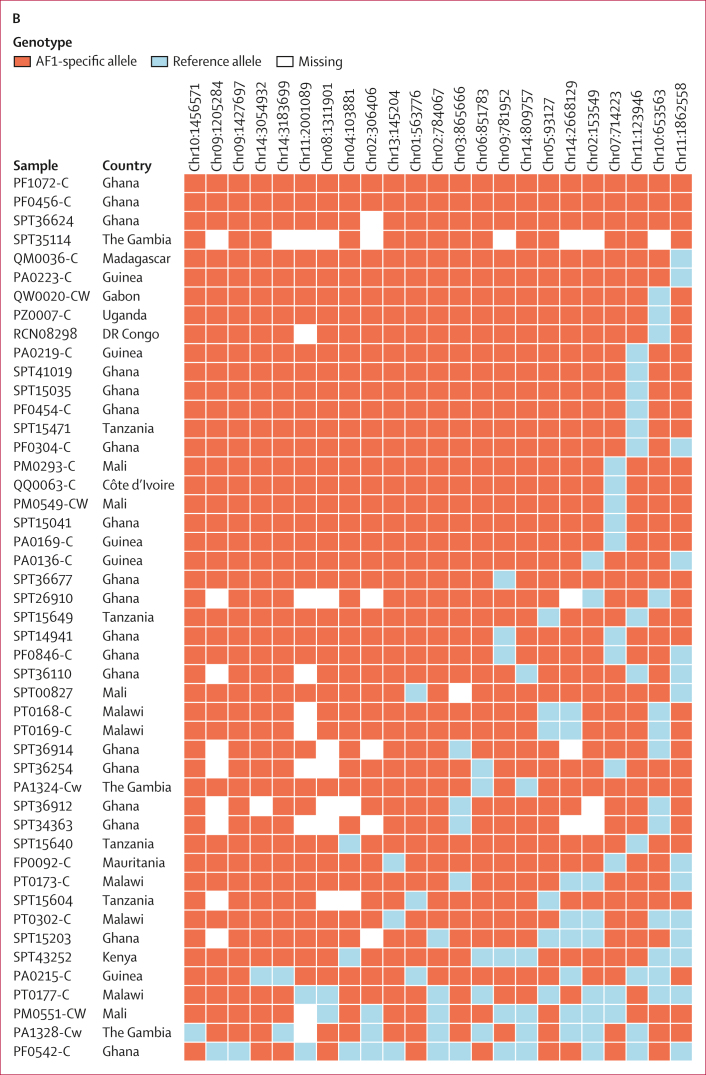

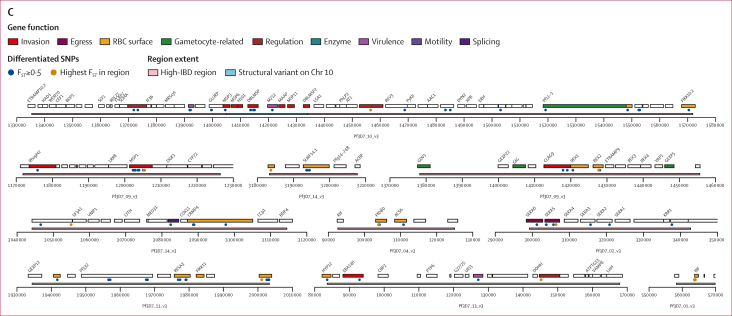
AF1 characteristic loci (A) The circular plot maps all 14 nuclear chromosomes (starting clockwise from the top, each chromosome is represented by a coloured segment in the outer ring). The inner ring shows a plot of mean *F*_*ST*_ between AF1 and the three African macroregions (west Africa, central Africa, and east Africa) at non-synonymous coding SNPs (appendix p 7). In high-IBD regions, at least 50% of all AF1 sample pairs are in IBD (appendix p 20). Internal lines show the *r*^*2*^ measure of linkage disequilibrium between pairs of high-*F*_*ST*_ SNPs (*F*_*ST*_>0·2), estimated using all African parasites. Three types of line represent three linkage disequilibrium ranges: *r*^*2*^ greater than or equal to 0·2, but less than 0·4; *r*^*2*^ greater than or equal to 0·4, but less than 0·5; and *r*^*2*^ greater than or equal to 0·5. (B) Presence of characteristic haplotypes in AF1 parasites. This panel shows a matrix of genotypes at each of the SNPs with the highest *F*_*ST*_ in the 23 high-IBD regions identified in the AF1 population. Each row represents an AF1 sample; the sample identification number and the country of provenance are shown. (C) Genes at AF1 characteristic loci. This panel shows maps of gene positions for the ten highest-ranked high-IBD regions identified in the AF1 population. The x-axis represents positions on the high-IBD region’s chromosome. Each gene in the region is shown by a rectangle, labelled with the gene’s name and coloured according to its function (when function is known). The highest-*F*_*ST*_ SNP in each region is detailed in the appendix (p 14). IBD=identity by descent. RBC=red blood cell. SNP=single nucleotide polymorphism.

Given the marked differentiation at the AF1 characteristic loci, we predicted a strong correlation between alleles found in these regions. This hypothesis was confirmed by computing *r*^*2*^, a commonly used linkage disequilibrium measure,^17^ for all distal pairs of SNPs with mean *F*_*ST*_ of at least 0·2. Several loci contained highly correlated distal SNPs (*r*^*2*^≥0·2); mapping these associations across the genome shows a complex linkage disequilibrium network (figure 3A). Seven differentiated loci each contained at least one SNP very strongly associated (*r*^*2*^≥0·4) with SNPs at all other loci (appendix p 14). Such strong associations provide clear evidence that AF1 parasites possess a multi-component genetic background, carried as a complete set by most members. However, determining the exact composition of this background will require further analysis, because high *r*^*2*^ values only occur when AF1 alleles are very rare outside AF1, which is not a requisite for a component locus.

### Ancestry analysis

To address the question of whether AF1 shared alleles originate from different sources in different countries, or have been co-inherited from common ancestry, we conducted an analysis of IBD for all sample pairs. This analysis identifies genomic regions in which parasites pairs are identical to an extent not explainable unless the two parasites have a common ancestry. AF1 parasites exhibited pairwise IBD at a much higher fraction of their genomes (median 22·4% [IQR 18–28]) than non-AF1 parasites in west Africa, central Africa, and east Africa (0·05% [0·00–0·79], 0·8% [0·00–1·30], and 1·2% [0·23–1·80], respectively; appendix p 18), suggesting that AF1 is differentiated by haplotypes with shared ancestries. This common ancestry was confirmed by PCoA, using a distance measure derived from IBD genome fractions (appendix p 19). Although pairwise IBD levels are well above those in other African populations, AF1 is not a clonally expanding population. Specifically, west African AF1 genomes shared significantly higher IBD fractions with west African genomes than with east African genomes (0·67% [0–1·30] *vs* 0·18% [0–0·69]), and vice versa (0·59% [0–1·10] *vs* 0 [0–0·73]; appendix p 18), indicating that recombination occurs between AF1 parasites and non-AF1 local populations.

Hypothesising that IBD is restricted to specific genomic regions, we mapped the frequency of IBD segments, identifying 23 regions in which more than 50% of all AF1 pairs were in IBD (appendix p 20). These high-IBD regions were present in all chromosomes except chromosome 12, often near subtelomeric regions. Each high-IBD region contained one or more SNPs with a mean *F*_*ST*_ of more than 0·5 and AF1 characteristic allele frequency of more than 0·5 (appendix p 14). The high-*F*_*ST*_ SNPs, ranked by allele frequency, are effective markers for identifying AF1 members: 42 (89%) of 47 members carry the AF1 characteristic alleles at all top seven ranked SNPs, and no more than one non-AF1 allele at the top 13 ranked SNPs (figure 3B). Conversely, only one non-AF1 sample carried AF1 alleles at more than half of the six top ranked SNPs, suggesting that AF1 members can be distinguished by simple genetic tests.

Taken together, results from analyses of IBD, differentiation, and correlation show that highly differentiated loci are mostly located in high-IBD regions and strongly linked across chromosomes (figure 3A). We can deduce that AF1 parasites carry a constellation of variants that differentiate them from other African parasites. These variants appear to be inherited together, even though AF1 genomes recombine with sympatric strains. It appears that not all the loci involved are equally important: most AF1 members carry a core set of approximately 13 characteristic haplotypes, although other loci seem to be less crucial components. All evidence suggests that the variant constellation is co-inherited, rather than having different ancestries in different countries.

### Structural variants in chromosome 10 and 9

**Figure 4:**
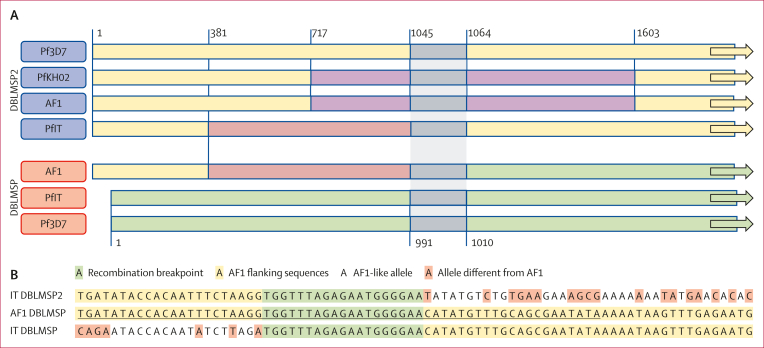
*DBLMSP* gene sequence crossover in AF1 parasites (A) Schematic of the gene conversion underpinning the AF1 variant of *dblmsp*. The diagram shows as colour blocks the sequences of *dblmsp* and *dblmsp2* in four *Plasmodium falciparum* genomes: Pf3D7 (reference), PfIT (long-read sequenced), PfKH02 (long-read sequenced), and AF1 (de novo assembly of sample PM0293-C). Blocks of the same colour indicate highly similar (near-identical) sequences. Coordinates shown (not to scale) correspond to the Pf3D7 positions in *dblmsp2* (above) and *dblmsp* (below). The AF1 *dblmsp* sequence is near-identical to that of PfIT *dblmsp2* at the 5′ end, and of PfIT *dblmsp* after position 991. The AF1 *dblmsp2* sequence, however, is near-identical to the *dblmsp2* sequence of PfKH02. The grey region is a 19-nucleotide sequence identical in *dblmsp* and *dblmsp2*, providing a recombination breakpoint. (B) Detail of the AF1 *dblmsp* and *dblmsp2* breakpoint region. This panel shows an alignment of the AF1 *dblmsp* sequence (middle) against the *dblmsp2* (above) and *dblmsp* (below) sequences of PfIT. The 19-nucleotide region of 100% identity is shown in green; to the left, the AF1 sequence is identical to PfIT *dblmsp2*, whereas to the right it is identical to PfIT *dblmsp*. The underlined 62-nucleotide portion of the AF1 sequence was used as search query to confirm the presence of the conversion breakpoint in the AF1 parasites.

The top-ranked high-IBD region on chromosome 10 (figure 3C) is also the largest of these regions. Due to its size, we hypothesised that this region could harbour a structural variant. Sequencing read coverage showed that AF1 members had few or no reads mapping to genes *msp6* (PF3D7_1035500) and *h101* (PF3D7_1035600), suggesting a large deletion (appendix p 21). The adjacent *dblmsp* gene (PF3D7_1035700) was also poorly covered at the 5′ end, but the presence of a proximal paralog (*dblmsp2*, PF3D7_1036300) raised the possibility of short read mismapping. To clarify, we performed de novo assembly (appendix p 3) of the sequencing reads of an AF1 member from Mali (PM0293-C), mapping the resulting contigs to multiple *dblmsp* and *dblmsp2* reference sequences. The AF1 *dblmsp* sequence shows marked sequence similarity to PfIT (a South American strain), but a very different organisation, being almost identical to the PfIT *dblmsp2* gene at the 5′ end (figure 4A). This structural difference suggests a gene conversion event, through which the AF1 *DBLMSP* gene acquired the 5′ portion of *dblmsp2*. The presence of this structural variant explains the absence of coverage in that segment when aligning against the *dblmsp* reference, which was confirmed by a realignment against the de novo assembled AF1 sequences (appendix p 22). We tested AF1 sequencing reads for a sequence containing the recombination breakpoint and flanking regions (figure 4B, appendix p 20), which confirmed the gene conversion in 42 (89%) of 47 samples. Both the gene conversion and the deletion of genes *msp6* and *h101* were also verified by long-read assembly of an AF1 parasite from a different study^18^ (appendix pp 3, 23–24). We observed that other genes in this region contain AF1 high-*F*_*ST*_ SNPs, including *msp3* (PF3D7_1035400) and the glutamate-rich protein gene (*glurp*, PF3D7_1035300).

The second-ranked high-IBD region, on chromosome 9 (figure 3C), exhibits a highly differentiated haplotype in the merozoite surface protein *msp1* gene (PF3D7_0930300). Low coverage in some *msp1* regions was observed when aligning AF1 reads against the 3D7 reference (appendix p 25), suggesting that the AF1 sequence differs substantially from that reference. *msp1* is known to consist of frequently recombining blocks, and has been classified based on the variants present in four blocks.^19^ Alignments against reference strains showed that PM0293-C has a mad20/k1/k1/k1 *msp1* sequence, uncommon in non-AF1 African populations, but more frequent in South America and southeast Asia (appendix p 7). The PM0293-C amino acid sequence is near-identical to that of PfHB3, a Mesoamerican strain. We validated this result by long-read resequencing of an amplicon spanning the entire *msp1* gene of an AF1 sample (appendix pp 4, 26), and by inspecting long-read assemblies from an earlier study (appendix p 4).^18^

### Functional analysis of AF1 characteristic loci

The large number of loci and the low frequency of the characteristic alleles in non-AF1 parasites suggest an extremely low probability of inheriting a full complement of AF1 alleles when recombining with non-AF1 parasites. Given that the complete allele constellation circulates at detectable frequency, it is likely to be under selective pressure, possibly due to a fitness advantage conferred by functionally related mutations. There are known relationships between the chromosome 10 and 9 loci: on the merozoite surface, Msp1 binds with other surface proteins, including Dblmsp, Dblmsp2, and Msp6.^20^ The resulting complex plays a crucial role in merozoite egress and invasion of erythrocytes, also involving Sera5 and Sera6, whose genes (PF3D7_0207600 and PF3D7_0207500, respectively) carry high-*F*_*ST*_ SNPs on chromosome 2.^21^^,^^22^ High-*F*_*ST*_ variants in genes involved in erythrocyte interaction were found at other AF1 characteristic loci, including those encoding other merozoite surface proteins (Msp7 and Msp10), several *phist* gene family members,^23^ and a number of genes encoding proteins involved in exporting to the erythrocyte membrane, such as Resa3 (PF3D7_1149200), PfD80 (PF3D7_0401800), Mahrp1 (PF3D7_1370300),^24^ Pf332 (PF3D7_1149000),^25^ and the ring-exported proteins Rex1 and Rex2 (PF3D7_0935900 and PF3D7_0936000, respectively).^26^^,^^27^ In addition, several genes encoding erythrocyte-exported proteins carry AF1 differentiated alleles (eg, members of the *fikk* and *surfin* families) and the cytoadherence-linked asexual gene *clag9* (PF3D7_0935800). Thus, several AF1 characteristic variants are associated with common functional categories (figure 3C). A functional enrichment analysis (appendix p 5) verified that significant proportions of AF1 high-*F*_*ST*_ variants are associated with host cell surface, surface binding, processes of erythrocyte invasion and egress, and interactions with the immune system and regulatory functions (appendix p 16). The evidence points to a constellation of variants that are functionally linked and related to host–parasite interactions.

## Discussion

The analyses presented in this Article, based on 3783 high-quality *P falciparum* genomes, identified a genetic background of remarkable complexity, circulating across the breadth of the African continent and maintaining its integrity without solely relying on inbreeding. To our knowledge, this is the first report of what we describe as a cryptotype. A cryptotype is a complex inherited genetic background that remains hidden within genomes that are otherwise similar to those of sympatric parasites. Unlike what is observed in clonally expanding populations,^5^ IBD is not evenly distributed across the AF1 genome, but concentrated in numerous distal regions. The cryptotype’s ability to retain identity at its characteristic loci, over the long period of time it must have taken to achieve its geographical spread, is hard to reconcile with the extremely low probability of retaining variant constellations intact through outbreeding. Therefore, it seems probable that the AF1 genomes are maintained through both frequent inbreeding and, far more rarely, acquisition of non-AF1 genes through outbreeding.

The fact that more than 20 identical AF1 variants are found in parasites from Madagascar, Ghana, and DR Congo suggests a fine-tuned functional interplay between these loci, and a phenotypic benefit of carrying the complete constellation. Such a functional benefit would help maintain AF1 at detectable frequencies by, for example, bestowing a selected fitness advantage, or by providing adaptation to a specific niche where AF1 is particularly competitive. Occupying an exclusive niche (eg, a particular vector species or host population) would provide some level of reproductive isolation, promoting inbreeding and helping maintain the variant constellation. Although at this point we cannot identify the functional advantage conferred by the cryptotype, we note that many AF1-differentiated variants are functionally related. Several of the genes encode proteins that participate in erythrocyte egress and invasion, or export of parasite antigens to the red blood cell surface. Taken together, these lines of evidence suggest that the AF1 variant ensemble underpins phenotypic changes related to host erythrocyte interactions. We hypothesise that AF1 parasites have adapted to a specific erythrocyte-related host niche, for example, a haemoglobinopathy that reduces invasion^28^ or prevents erythrocyte remodelling.^29^ Although the broad geographical distribution makes it unlikely that the cryptotype is fine-tuned to a specific human population, it is possible that its evolutionary niche involves a non-human host.

Our analysis opens several questions that will require further investigation. Culturing in vitro field isolates can elucidate the biological mechanisms underpinning the cryptotype and the properties conferring its selective advantage, and provide material for high-quality, high-coverage long-read sequencing to investigate structural rearrangements. Identifying patients infected with AF1 parasites might help characterise the cryptotype’s evolutionary niche and understand its epidemiology. Given AF1’s low prevalence, such studies will be challenging but could produce important shifts in our understanding of invasion mechanisms and of protective human blood phenotypes. The wide-ranging catalogue of variants identified in this study can already provide experimental parasitologists with candidates for studying gene interactions and synergies.

A question emerging from this work is whether AF1 is the sole cryptotype in Africa, or whether other complex genetic backgrounds circulate in this or other continents. AF1 parasites separate clearly in PCoA plots largely because their differentiated variants are mostly absent from other African populations, resulting in high levels of differentiation. However, the absence of characteristic alleles from the general population is not a requisite for cryptotypes. Clusters of individuals carrying co-inherited variants could be difficult to detect by PCoA when these variants are common outside the clusters. Alternative approaches might be needed, for example, those based on sensitive IBD detection algorithms. Furthermore, detecting cryptotypes at a low frequency could require larger genomic datasets. We have shown that analysing genomic data shared by a multitude of studies can lead to important discoveries. We advocate that repositories providing such data in organised and usable forms, such as those managed by MalariaGEN,^6^ must continue to be supported by funders and contributing researchers alike, to power advancements in understanding of epidemiological phenomena.

## Data sharing

Sequencing data used in the present study are publicly available as part of the open-access MalariaGEN Pf7 dataset.

## Declaration of interests

We declare no competing interests.
