# Supplementary material for: Identification of complex Plasmodium falciparum genetic backgrounds circulating in Africa: a multicountry genomic epidemiology analysis
Source: Lancet Microbe. 2024 Dec;5(12):None. doi: 10.1016/j.lanmic.2024.07.004 (PMC11628469; doi:10.1016/j.lanmic.2024.07.004)
Supplement: Supplementary appendix [file mmc1.pdf]

THE LANCET
Microbe

Supplementary appendix

This appendix formed part of the original submission and has been peer reviewed.
We post it as supplied by the authors.

Supplement to: Miotto O, Amambua-Ngwa A, Amenga-Etego LN, et al. Identification of complex *Plasmodium falciparum* genetic backgrounds circulating in Africa: a multicountry genomic epidemiology analysis. *Lancet Microbe* 2024. https://doi.org/10.1016/j.lanmic.2024.07.004

Identification of complex *Plasmodium falciparum* genetic backgrounds circulating in Africa: a multi-country genomic epidemiology analysis

Miotto O et al.

Supplementary Materials

Contents

Supplementary Text 2
Selection of Samples and Variants 2
Identification of high-quality SNPs in non-sWGA samples 2
Linkage Disequilibrium (*r^2*) calculations 2
Confirmation of DBLMSP/DBLMSP2 recombination 3
Validation of DBLMSP/DBLMSP2 de novo assemblies by short read alignment. 3
Validation of AF1 variants by long-read sequences 3
Validation of Chromosome 10 variants by long-read sequences 3
Validation of MSP1 by long-read sequence assembly 4
Validation of MSP1 by long-read amplicon sequencing 4
Functional Enrichment Analysis 5
Supplementary Tables 6
Supplementary Table 1 – Summary of sample counts by year. 6
Supplementary Table 2 – Highly Differentiated non-synonymous coding SNPs in AF1. 7
Supplementary Table 3 – Correlation between AF1 characteristic loci. 14
Supplementary Table 4 – High-IBD genomic regions in the AF1 group 15
Supplementary Table 5 – Functional Enriched Gene Ontology (GO) Terms in genes carrying SNPs highly differentiated in AF1. 16
Supplementary Figures 17
Supplementary Figure 1 – Genome-wide map of F_{ST} between AF1 and other African populations. 17
Supplementary Figure 2 – Pairwise IBD fraction levels within and between African populations. 18
Supplementary Figure 3 – PCoA plot based on an IBD distance measure. 19
Supplementary Figure 4 – Genome-wide map of pairwise IBD within AF1. 20
Supplementary Figure 5 – Coverage of the Chromosome 10 locus in an AF1 sample. 21
Supplementary Figure 6 – Coverage profiles of AF1 sequencing read alignments on predicted *de novo* assembly reads. 22
Supplementary Figure 7 – Long-read confirmation of a large deletion at the AF1 Chromosome 10 locus. 23
Supplementary Figure 8 – Long-read confirmation of the DBLMSP/DBLMSP2 gene conversion at the AF1 Chromosome 10 locus. 24
Supplementary Figure 9 – Alignment of AF1 sequencing reads in the MSP1 gene. 25
Supplementary Figure 10 – Alignment of AF1 long sequencing reads against two different MSP1 gene references. 26
References for Supplementary Materials 27

SUPPLEMENTARY TEXT

Selection of Samples and Variants

In our selection of samples and variants to be analyzed, we focussed on reducing genotype missingness, i. e. the proportion of genotypes that could not be called due to insufficient sequencing read coverage. High missingness significantly affects analyses such as genetic distance estimation, identity by descent (IBD), correlations, allele frequency, etc.

Taking the complete Pf7 dataset (20,864 samples) as a starting point, we extracted genotypes for at 2,211,959 single nucleotide polymorphisms (SNPs) that were biallelic and labelled as "PASS" in the release data. After removing samples with >50% of missing genotypes, we retained 2,025,136 *typable SNPs* with had genotypes in ≥75% of the remaining samples. Similarly, we identified the set of 15,051 *typable samples*, which had a valid genotype at ≥75% of typable SNPs. The samples were further filtered, retaining only those labelled as "QCpass=TRUE" in the Pf7 dataset, to remove duplicate samples and laboratory strains.

For our analyses, we wished to use only samples collected in Africa, and *essentially monoclonal* since multiclonal samples are not amenable to IBD analyses. In addition, we wished to remove SNPs that do not show variability in Africa. Using Pf7 metadata, we selected samples from African countries that had F_{WS}≥0.95, deriving a final sample set of 3,783 samples in three macroregions: West, Central and East Africa (WAF, CAF and EAF respectively, Table 1). After estimating minor allele frequency (MAF) in each of these macroregions for every typable SNP, we discarded SNPs with MAF<0.001 and those that did not have at least one sample with a homozygous genotype for each allele. This reduced the set of variants to 743,584 typable SNPs, that were used in our analyses.

Identification of high-quality SNPs in non-sWGA samples

Many of the more recent genome sequences in the Pf7 dataset were obtained by *selective whole-genome amplification* (sWGA), a laboratory protocol that enables whole-genome sequencing from dried blood spots.[1] Although this process has been critical for the growth of the genomic dataset, it produces an uneven amplification profile, causing some genome segments to be poorly covered in sWGA. As a result, some SNPs that are well-covered in non-sWGA processed samples show high genotype missingness in sWGA samples, leading to these SNPs being discarded from the analysis set. We observed that some of these discarded SNPs were located in the AF1 characteristic regions, and wished to analyze them since they could be highly differentiated in AF1. To obviate this problem, we selected the subset of samples that were not processed by sWGA prior to sequencing (n=1,829 in WAF, CAF and EAF), and repeated the SNP filtering and F_{ST} estimation procedures on this sample set. This produced an additional set of 68,360 SNPs that could be genotyped in non-sWGA samples. These SNPs were only used in the identification of AF1 highly differentiated variants.

Linkage Disequilibrium (r^2) calculations

The linkage disequilibrium measure r^2 between two SNPs S_1 and S_2 was calculated from the allele frequencies of the major and minor allele at S_1 (p_1 and p_2 respectively) and S_2 (q_1 and q_2), and the *allele pair frequencies* x_{11}, x_{21}, x_{12}, x_{22} (the indices indicating whether the major or minor allele is present at each of the two SNPs) as follows: $r^2 = D^2/(p_1 p_2 q_1 q_2)$ where $D = x_{11} - p_1 q_1$.[2] Only SNPs with mean F_{ST}≥0.2 were used in linkage pairs. The circular genome linkage disequilibrium plot was generated using circos v0.69.[3]

Confirmation of DBLMSP/DBLMSP2 recombination

In the AF1 DBLMSP gene sequence, we located the join between the 5' portion acquired from the DBLMSP2 gene, and the 3' DBLMSP portion. At this locus, we identified a 19-nt stretch which is identical in the two genes, and likely to be the recombination breakpoint (Figure 4B). We derived a 62-nt sequence, comprising this breakpoint segment and two flanking fragments, which was used as a search template to confirm the presence of the DBLMSP gene conversion, by inspecting the sequencing reads of AF1 members.

Matches for the sequence were found in 42 out of 47 AF1 samples; in the remaining samples we could not identify the DBLMSP sequence, possibly because of localized poor coverage, or of an alternative structural variant.

Validation of DBLMSP/DBLMSP2 de novo assemblies by short read alignment.

To confirm the validity the *de novo* assembled sequences of genes DBLMSP and DBLMSP2 derived from short reads from AF1 sample PM0293-C, we separately used each of the two assemblies as reference for an alignment of short reads from AF1 samples. The alignments were conducted separately to avoid alignment competition (i.e. the same reads may have been mapped in both alignments). Four AF1 samples were used: PM0293-C, PA0136-C, PF1072-C and PT173-C. The resulting alignments were visualized using the Integrative Genomics Viewer (IGV)[4]. We found that all alignments showed even coverage without sizeable coverage gaps, over most of the coding sequence (Supplementary Figure 6). This is in contrast with the lack of coverage in the 5' regions of DBLMSP when aligning against the Pf3D7 reference (Supplementary Figure 5). The lack of coverage gaps is strong evidence that the two assemblies correspond to the genomes that generated the short reads, and therefore consistent with a correct assembly of the AF1 sequences.

Validation of AF1 variants by long-read sequences

Through a search in the NCBI public databases using the BLAST tools (https://blast.ncbi.nlm.nih.gov/) we identified whole-genome data for a single *Pf* sample carrying a set of AF1 mutations. These data had been submitted with a previous publication by Antoine Dara and colleagues.[5] The sample (NCBI Biosample accession code: SAMN02373819, study code: 318.1) was the only one carrying AF1 alleles amongst 12 samples collected by the study in Mali in 2010. It was sequenced using both Illumina short-read (accession numbers: SRX363955 and SRX363957) and PacBio long-read (accession numbers: SRX363953, SRX363954, SRX363956 and SRX363958-SRX363961) sequencing technologies. Genomic assemblies were then created by constructing contigs from the PacBio reads, and using aligned Illumina reads to correct errors and improve contig quality. The contigs were also made available (accession numbers: GCA_001861165.1).

To confirm that the parasites in the sample belong to the AF1 group, we aligned the 318.1 contigs to the Pf3D7_v3 reference genome, and used `bcftools pileup`[6] to call the 23 characteristic AF1 variants (high-IBD region markers, see Supplementary Table 3) identified in the present manuscript. The sample was found to carry 21/23 AF1 mutations, including the 6 most common variants, and was therefore confirmed as AF1.

Validation of Chromosome 10 variants by long-read sequences

To confirm the structural variants predicted in the Chromosome 10 region containing the DBLMSP/DBLMSP2 genes, we identified two large contigs that cover the region in question (contig IDs: JPSB01001330.1 and JPSB01001338.1). For each contig, we created an alignment against the

Pf3D7_v3 reference genome, as well as an alignment of the PacBio long reads against the contig; alignments were performed using minimap2.[7] The JBrowse2 genome browser[8] was then used to create synteny plots that show the two alignments simultaneously.

The plot for contig JPSB01001330.1 showed evidence of a ~19kb deletion (approximate coordinates: 1,408k -1,427k) which contained genes *MSP6* (Pf2D7_1035500), *H101* (Pf2D7_1035600), *DBLMSP* (Pf2D7_1035700), *M721* (Pf2D7_1035800) and *M556* (Pf2D7_1035900); the long reads spanning the deletion confirm that it is not an artefact of short-read assemblies (Supplementary Figure 9).

The plot for contig JPSB01001338.1 showed a more complex structural variant (Supplementary Figure 10). The first portion of the contig (~0-3.9 kbp) mapped to the 5' end of BDLMSP2 (Pf2D7_1036300) and its preceding non-coding region (approximate coordinates: 1,428.8k - 1,433.2k), while the remainder of the contig (~3.2-16.4 kbp) mapped from the 5' portion of BDLMSP1 (Pf2D7_1035700) to the 3' portion of MSP1 (Pf2D7_1036000) (approximate coordinates: 1,413.6k -1,427.6k), encompassing genes *M721* (Pf2D7_1035800) and *M556* (Pf2D7_1035900). The two portions of the contig overlap in a region (~3.6-3.9 kbp) that to maps to a central region of both DBLMSP and DBLMSP2 (Figure 4); several long reads span this region and go well into the portions of the contig on either side, demonstrating that the structural variant is not an artefact of the assembly. Taken together, these results confirmed the *de novo* assemblies from Illumina data, and specifically: (a) the gene conversion that has taken place between *DBLMSP* and *DBLMSP2*; (b) the deletion of genes *MSP6* and *H101*; and (c) the duplication of *MSP11*. It is as yet unclear in which order the two contigs appear in the AF1 genome, or indeed whether they occur near each other.

Validation of MSP1 by long-read sequence assembly

To confirm the predicted MSP1 sequence, we identified a contig in sample 318.1 that covers the whole gene (contig ID: JPSB01001312.1). When aligning the gene sequence from this contig to the sequence assembled from short reads generated from sample PM0293-C, as well as those from the reference strains PfIT and PfHB3, we found near-identity over the whole gene length. The only substantial differences were a 9-bp insertion in the repetitive Block 2, and a 24-bp insertion in the highly variable Block 8. The contig therefore confirms that the MSP1 gene in the AF1 population has the same structure as that of PfIT and PfHB3, with a MAD20/K1/K1/K1 signature.

Validation of MSP1 by long-read amplicon sequencing

In a further experiment to confirm the sequence of the MSP1 gene in AF1 parasites, we obtained a stored DNA aliquot for one of the AF1 samples analysed here (sample SPT15471 from Tanzania), and amplified a genomic segment containing the complete MSP1 gene using a single PCR reaction (forward primer: AGAAGATGCAGTATTGACAGGT; reverse primer: GAACTGCAGAAAATACCATCGA). The amplicons were sequenced on an Oxford Nanopore Technologies (ONT) MinION mk1b device with R10.4.1 flow cells using native barcoding kit NBD114.24, as previously described.[9] ONT sequencing is able to sequence the full length of the amplicons, thus avoiding read assemblies. We used minimap2 to align the resulting amplicon sequences against two different references: the 3D7 and HB3 genomes. Inspection of the alignments (Supplementary Figure 12) revealed that, after filtering for sequencing errors, the AF1 MSP1 sequence is essentially identical to that of HB3, while it diverges very markedly from the 3D7 sequence in most of the MSP1 gene. This is not unexpected, given the extreme polymorphism of MSP1, and explains the uneven coverage produced by short

reads (Supplementary Figure 11); the ONT long sequences, on the other hand, cover some MSP1 segments that are sufficiently similar to allow read mapping.

Functional Enrichment Analysis

To provide statistical support for the functional role of the AF1 genetic background components, we performed a Functional Enrichment Analysis of the genes carrying highly differentiated SNPs in the AF1 population. This used Gene Ontology (GO) term annotations for all Pf genes, retrieved from the PlasmoDB Pf GO annotations Release 68 (https://plasmodb.org/plasmo/app/downloads). Each category of GO terms ("biological process", "molecular function" and "cellular component") was processed separately as follows.

We extracted a list of all terms associated with at least 5 genes ("all terms"), as well as the list of their associated genes ("annotated genes"). We constructed a list of genes that contain at least one SNP with mean F_{ST}≥0.5 (n=107, Supplementary Table 2) and are present in the "annotated genes" list ("AF1 genes"); the remaining members of the "annotated genes" formed a "Non-AF1 genes" list. We extracted all GO terms associated with the "AF1 genes", and for each GO term we determined the proportions of "AF1 genes" and "Non-AF1 genes" associated to this term. We performed a one-sided Fisher's Exact Test, applying a Benjamini-Hochberg False Discovery Rate (FDR) correction to the resulting p-values, to find terms for which the proportion of "AF1 genes" was significantly higher.

SUPPLEMENTARY TABLES

Supplementary Table 1 – Summary of sample counts by year.

Each row represents one year during which ≥20 samples included in our analyses were collected.
The columns show: the year; the total number of analysed samples collected in that year; the number of AF1 samples collected in that year, their percentage of the samples analysed (with 95% confidence interval), and the p-value of a Fisher's exact test comparing the proportion within the year against the proportion in the rest of the dataset (p<0.01 shown in bold type). Only 2011 showed significant differences in AF1 proportion; this is the year in which AF1 samples were collected in Guinea and Malawi (the two countries whose AF1 proportion is significantly different from the rest).

Year	Sample Count	AF1 Count	AF1 %	95% C.I.	*p*
**1984**	26			[0.0%, 15.2%]	1.00
**1994**	23			[0.0%, 16.9%]	1.00
**2005**	20			[0.0%, 19.0%]	1.00
**2006**	23			[0.0%, 16.9%]	1.00
**2007**	67			[0.0%, 6.5%]	1.00
**2008**	78			[0.0%, 5.6%]	1.00
**2009**	70			[0.0%, 6.2%]	1.00
**2010**	159	3	1.9%	[0.4%, 5.7%]	0.45
**2011**	243	13	5.3%	[3.1%, 9.0%]	**<0·0001**
**2012**	167			[0.0%, 2.7%]	0.27
**2013**	684	6	0.9%	[0.4%, 1.9%]	0.44
**2014**	716	10	1.4%	[0.7%, 2.6%]	0.71
**2015**	386	4	1.0%	[0.3%, 2.7%]	1.00
**2016**	431	3	0.7%	[0.1%, 2.1%]	0.36
**2017**	320	2	0.6%	[0.0%, 2.4%]	0.43
**2018**	300	5	1.7%	[0.6%, 4.0%]	0.42
**Total**	**3,713**	**46**	**1.2%**	**[0.9%, 1.7%]**	

Supplementary Table 2 – Highly Differentiated non-synonymous coding SNPs in AF1.

Each row represents one non-synonymous coding SNP (n=198) that exhibits mean $F_{ST} \geq 0.5$ between AF1 and the three African macro-regions. The columns show: chromosome number and position of the SNP within the chromosome; mean F_{ST} and standard deviation; ID and description of the gene containing the SNP; the amino acid mutation caused by the non-reference allele; and the estimated frequencies of the non-reference allele in AF1 and the following populations: West Africa (WAF), Central Africa (CAF), East Africa (EAF), South Asia (SAS), Western Southeast Asia (WSEA), Eastern Southeast Asia (ESEA), Oceania (OCE) and South America (SAM). SNPs with F_{ST} ≥0.75 are highlighted in **bold** type. Rectangular boxes enclose sets of SNPs that are found in the same AF1 high-IBD genomic regions (see Supplementary Figure 4). SNPs that had high missingness only in sWGA-processed samples (see main text and Methods) are indicated by a coloured background in their Chr/Pos fields. Reported positions, identifiers, mutations and non-reference alleles are all with respect to the 3D7 V3 reference genome. To aid visualization, the backgrounds of the F_{ST} and frequency estimates were coloured so that higher values are represented by more saturated background colours.

Chr	Pos	Mean F_{ST}	SD F_{ST}	Gene ID	Gene Description	Mutation	AF1	WAF	CAF	EAF	SAS	WSEA	ESEA	OCE	SAM
1	114559	0.62	0.046	PF3D7_0102500	erythrocyte binding antigen-181	N414I	0.82	0.03	0.02	0.08	0.72	0.99	0.99	0.78	0.08
1	114724	0.66	0.017			R359K	0.81	0.01	0.00	0.02	0.03	0.34	0.31	0.59	0.08
1	132574	0.52	0.0078	PF3D7_0103000	vacuolar protein sorting-associated protein VTA1, putative	E182K	0.81	0.10	0.09	0.09	0.02	0.00	0.00	0.00	0.00
1	180034	0.63	0.013	PF3D7_0104100	protein E140, putative	E540D	0.09	0.88	0.89	0.88	0.86	0.85	0.87	0.88	0.47
1	527210	0.55	0.015	PF3D7_0113800	DBL containing protein, unknown function	N35S	0.83	0.10	0.07	0.09	0.07	0.02	0.00	0.08	0.01
**1**	**563776**	**0.80**	0.015	**PF3D7_0114700**	**PIR protein**	**A300V**	0.93	0.03	0.05	0.03	0.01	0.00	0.00	0.00	0.04
2	153549	0.65	0.0033	PF3D7_0203100	protein kinase, putative	E1145K	0.80	0.01	0.01	0.01	0.00	0.00	0.00	0.00	0.00
**2**	**301238**	**0.89**	0.0058	**PF3D7_0207500**	**serine repeat antigen 6**	**Q290K**	0.95	0.00	0.00	0.01	0.00	0.00	0.00	0.00	0.00
**2**	**303754**	**0.90**	0.0052	**PF3D7_0207600**	**serine repeat antigen 5**	**K945E**	0.95	0.00	0.00	0.01	0.00	0.00	0.00	0.00	0.00
**2**	**303786**	**0.90**	0.005			**R934H**	0.95	0.00	0.00	0.01	0.00	0.00	0.00	0.00	0.00
**2**	**305718**	**0.88**	0.034			**I330L**	0.95	0.00	0.00	0.04	0.75	1.00	0.99	0.93	1.00
**2**	**306406**	**0.85**	0.019			**K159E**	0.93	0.00	0.00	0.02	0.79	1.00	1.00	0.92	1.00
2	315716	0.59	0.027	PF3D7_0207800	serine repeat antigen 3	T234P	0.05	0.83	0.81	0.78	0.89	1.00	0.99	1.00	0.91
2	320853	0.67	0.061	PF3D7_0207900	serine repeat antigen 2	P89S	1.00	0.24	0.14	0.22	0.00	0.00	0.00	0.02	0.00
2	337669	0.56	0.014	PF3D7_0208300	conserved Plasmodium protein, unknown function	Y38N	0.75	0.03	0.01	0.02	0.01	0.00	0.00	0.00	0.00
2	373697	0.52	0.11	PF3D7_0209000	transmission-blocking target antigen s230	S1087Y	0.86	0.25	0.14	0.05	0.28	0.17	0.08	0.00	0.10
2	735613	0.62	0.036	PF3D7_0217900	thioesterase/thiol ester dehydrase-isomerase, putative	S462T	0.81	0.06	0.01	0.03	0.00	0.00	0.00	0.00	0.00
**2**	**784067**	**0.79**	0.0015	**PF3D7_0219700**	**gametocyte exported protein 20**	**Y182H**	0.89	0.01	0.01	0.01	0.00	0.00	0.00	0.00	0.15
2	784379	0.64	0.05			H78N	0.92	0.16	0.11	0.08	0.15	0.07	0.02	0.00	0.15
2	814192	0.57	0.002	PF3D7_0220300	Plasmodium exported protein, unknown function	P92A	0.72	0.00	0.00	0.00	0.00	0.00	0.00	0.00	0.00
3	865666	0.63	0.02	PF3D7_0320700	signal peptidase complex subunit 2	M78L	0.89	0.08	0.09	0.11	0.00	0.00	0.00	0.00	0.48

Chr	Pos	Mean F_{ST}	SD F_{ST}	Gene ID	Gene Description	Mutation	AF1	WAF	CAF	EAF	SAS	WSEA	ESEA	OCE	SAM
**4**	**103881**	0.85	0.016	**PF3D7_0401800**	**Plasmodium exported protein (PHISTb), unknown function**	**K515R**	0.93	0.00	0.01	0.02	0.41	0.84	0.68	0.71	0.00
4	103987	0.53	0.042			K480E	0.04	0.74	0.72	0.80	0.77	0.89	0.57	0.97	0.69
4	104157	0.58	0.072			H423P	0.91	0.09	0.20	0.17	0.21	0.13	0.33	0.14	0.00
4	110821	0.57	0.056	PF3D7_0401900	acyl-CoA synthetase	I159L	0.98	0.20	0.30	0.23	0.59	0.82	0.47	0.70	0.11
**4**	**464779**	0.79	0.018	**PF3D7_0410000**	**erythrocyte vesicle protein 1**	**D819Y**	0.90	0.02	0.00	0.03	0.00	0.00	0.00	0.00	0.05
4	1103709	0.58	0.026	PF3D7_0424400	surface-associated interspersed protein 4.2 (SURFIN 4.2)	W1247.	0.76	0.02	0.04	0.00	0.00	0.00	0.00	0.00	0.00
4	1113576	0.61	0.097	PF3D7_0424600	Plasmodium exported protein (PHISTb)	K233N	0.89	0.05	0.08	0.20	0.68	0.69	0.51	0.95	0.51
6	851783	0.59	0.11	PF3D7_0620400	merozoite surface protein 10	K391N	0.89	0.09	0.07	0.24	0.10	0.02	0.01	0.00	0.43
7	712688	0.58	0.012	PF3D7_0716200	PDCD2 domain-containing protein, putative	G74R	0.74	0.02	0.00	0.00	0.00	0.00	0.00	0.00	0.00
7	1359488	0.65	0.075	PF3D7_0731500	erythrocyte binding antigen-175	K478N	0.90	0.05	0.08	0.17	0.07	0.07	0.22	0.38	0.08
8	1056829	0.68	0.081	PF3D7_0824200	conserved Plasmodium protein, unknown function	L474I	0.07	0.93	0.92	0.81	0.71	0.63	0.42	0.62	0.88
8	1238850	0.60	0.006	PF3D7_0828800	GPI-anchored micronemal antigen	V218I	0.79	0.03	0.02	0.02	0.00	0.00	0.00	0.00	0.00
8	1296885	0.59	0.03	PF3D7_0830500	tryptophan-rich antigen	F426Y	0.82	0.05	0.05	0.08	0.60	0.83	0.83	0.77	0.26
8	1311901	0.68	0.032	PF3D7_0830800	surface-associated interspersed protein 8.2 (SURFIN 8.2)	P422R	0.95	0.14	0.10	0.14	0.13	0.09	0.13	0.06	0.03
8	1311927	0.53	0.027			N431H	0.98	0.27	0.24	0.30	0.45	0.56	0.57	0.32	0.53
8	1311929	0.63	0.014			N431K	0.95	0.15	0.17	0.17	0.25	0.26	0.25	0.22	0.20
8	1311938	0.61	0.022			F434L	0.95	0.16	0.18	0.19	0.27	0.26	0.25	0.22	0.20
8	1311959	0.52	0.029			L441F	0.95	0.23	0.23	0.28	0.30	0.41	0.32	0.32	0.21
8	1312185	0.62	0.046			N517H	0.95	0.13	0.21	0.18	0.11	0.24	0.17	0.11	0.03
8	1344521	0.66	0.23	PF3D7_0831400	Plasmodium exported protein, unknown function	N265D	0.98	0.46	0.06	0.09	0.63	0.82	0.96	0.67	0.50
8	1344529	0.66	0.23			I262K	0.98	0.45	0.06	0.09	0.62	0.82	0.96	0.67	0.50
**9**	**82156**	0.76	0.058	**PF3D7_0901700**	**Plasmodium exported protein (hyp5), unknown function**	**Y177C**	0.91	0.01	0.02	0.09	0.08	0.18	0.28	0.25	0.40
9	82238	0.63	0.094			N150D	0.93	0.07	0.14	0.23	0.21	0.37	0.36	0.36	0.40
9	84790	0.66	0.038	PF3D7_0901800	Plasmodium exported protein, unknown function	F56S	0.91	0.07	0.12	0.12	0.11	0.15	0.35	0.00	0.37
9	85450	0.51	0.097			T217N	0.91	0.13	0.19	0.32	0.41	0.72	0.78	0.66	0.53
**9**	**465933**	0.80	0.0088	**PF3D7_0910200**	**conserved Plasmodium protein, unknown function**	**T459A**	0.91	0.01	0.02	0.02	0.00	0.00	0.00	0.00	0.00
9	527158	0.53	0.025	PF3D7_0911500	conserved Plasmodium protein, unknown function	C147F	0.81	0.07	0.09	0.11	0.02	0.00	0.00	0.00	0.00
9	778894	0.68	0.01	PF3D7_0918900	gamma-glutamylcysteine synthetase	N446S	0.84	0.03	0.02	0.02	0.00	0.00	0.00	0.00	0.00
**9**	**781952**	0.75	0.0005	**PF3D7_0919000**	**nucleosome assembly protein**	**I76V**	0.87	0.01	0.01	0.01	0.01	0.06	0.05	0.36	0.00
9	799189	0.65	0.02	PF3D7_0919500	major facilitator superfamily domain-containing protein, put.	E231V	0.87	0.07	0.07	0.04	0.00	0.00	0.00	0.00	0.00
**9**	**1175905**	0.83	0.01	**PF3D7_0929400**	**high molecular weight rhoptry protein 2**	**A235T**	0.93	0.02	0.02	0.03	0.00	0.00	0.00	0.00	0.06

Chr	Pos	Mean F_{ST}	SD F_{ST}	Gene ID	Gene Description	Mutation	AF1	WAF	CAF	EAF	SAS	WSEA	ESEA	OCE	SAM
**9**	**1202267**	0.89	0.017			**F152L**	0.95	0.00	0.00	0.02	0.04	0.27	0.11	0.15	0.10
**9**	**1202292**	0.85	0.017			**E161Q**	0.93	0.00	0.00	0.02	0.04	0.27	0.11	0.15	0.10
**9**	**1202596**	0.93	0.016			**T262K**	0.97	0.00	0.01	0.02	0.04	0.33	0.11	0.04	0.10
**9**	**1202605**	0.92	0.013			**A265E**	0.97	0.01	0.01	0.02	0.04	0.33	0.11	0.04	0.10
**9**	**1202649**	0.92	0.015			**Q280K**	0.97	0.00	0.01	0.02	0.04	0.27	0.11	0.05	0.10
**9**	**1202664**	0.83	0.026			**D285N**	0.97	0.04	0.08	0.06	0.08	0.27	0.11	0.05	0.31
**9**	**1202665**	0.83	0.026			**D285A**	0.97	0.04	0.08	0.06	0.08	0.27	0.11	0.05	0.31
**9**	**1202669**	0.83	0.026			**N286K**	0.97	0.04	0.08	0.06	0.08	0.27	0.11	0.05	0.31
**9**	**1202913**	0.93	0.016			**K368E**	0.97	0.00	0.00	0.02	0.13	0.29	0.13	0.15	0.10
9	1203652	0.61	0.11			L614R	1.00	0.26	0.34	0.14	0.07	0.05	0.04	0.19	0.02
**9**	**1203952**	0.96	0.019			**S714N**	1.00	0.03	0.01	0.02	0.00	0.00	0.00	0.00	0.00
**9**	**1205118**	0.93	0.016			**H1103N**	0.97	0.00	0.00	0.02	0.04	0.29	0.11	0.16	0.24
**9**	**1205120**	0.93	0.016			**H1103Q**	0.97	0.00	0.00	0.02	0.04	0.29	0.11	0.16	0.10
**9**	**1205121**	0.93	0.016	**PF3D7_0930300**	**merozoite surface protein 1**	**N1104H**	0.97	0.00	0.00	0.02	0.04	0.29	0.11	0.16	0.10
**9**	**1205151**	0.91	0.0035			**N1114Y**	0.96	0.00	0.00	0.00	0.00	0.00	0.00	0.00	0.10
**9**	**1205284**	0.93	0.016			**V1158E**	0.97	0.00	0.00	0.02	0.04	0.28	0.10	0.14	0.10
**9**	**1205314**	0.93	0.016			**N1168S**	0.97	0.00	0.00	0.02	0.04	0.29	0.11	0.14	0.10
**9**	**1205324**	0.93	0.016			**K1171N**	0.97	0.00	0.00	0.02	0.04	0.29	0.10	0.14	0.10
**9**	**1205329**	0.93	0.016			**R1173K**	0.97	0.00	0.00	0.02	0.04	0.29	0.10	0.14	0.10
**9**	**1205343**	0.93	0.016			**I1178L**	0.97	0.00	0.00	0.02	0.04	0.29	0.10	0.14	0.10
**9**	**1205355**	0.93	0.016			**L1182F**	0.97	0.00	0.00	0.02	0.04	0.28	0.10	0.13	0.10
**9**	**1205360**	0.93	0.016			**N1183K**	0.97	0.00	0.00	0.02	0.04	0.29	0.10	0.13	0.10
**9**	**1205370**	0.93	0.016			**H1187N**	0.97	0.00	0.00	0.02	0.04	0.29	0.10	0.14	0.10
**9**	**1205377**	0.93	0.016			**G1189E**	0.97	0.00	0.00	0.02	0.04	0.29	0.10	0.14	0.10
**9**	**1205395**	0.93	0.016			**F1195Y**	0.97	0.00	0.00	0.02	0.04	0.28	0.10	0.14	0.10
**9**	**1205424**	0.93	0.016			**T1205A**	0.97	0.00	0.00	0.02	0.04	0.29	0.11	0.14	0.10
9	1316936	0.53	0.084	PF3D7_0933100	conserved Plasmodium protein, unknown function	V606A	0.91	0.12	0.19	0.28	0.05	0.00	0.00	0.01	0.95
**9**	**1417854**	0.80	0.074	**PF3D7_0935800**	**cytoadherence linked asexual protein 9**	**T779S**	0.97	0.15	0.06	0.05	0.00	0.00	0.00	0.00	0.00
9	1419023	0.74	0.019			K1098Q	0.97	0.11	0.14	0.11	0.32	0.21	0.31	0.50	0.87
9	1420566	0.68	0.022	PF3D7_0935900	ring-exported protein 1	E687Q	0.84	0.03	0.04	0.01	0.00	0.00	0.00	0.00	0.04
**9**	**1427697**	0.90	0.031			**S77.**	0.98	0.06	0.02	0.02	0.00	0.00	0.00	0.00	0.32
**9**	**1427982**	0.88	0.031	**PF3D7_0936000**	**ring-exported protein 2**	**E14A**	0.98	0.06	0.02	0.03	0.02	0.31	0.07	0.10	0.86
9	1428013	0.70	0.061			Y4N	0.98	0.11	0.14	0.21	0.47	0.68	0.44	0.36	0.88
10	354221	0.60	0.032	PF3D7_1008500	protein GPR89, putative	L840P	0.96	0.18	0.18	0.23	0.19	0.06	0.01	0.00	0.00
10	559189	0.50	0.042	PF3D7_1014100	merozoite surface protein MSA180	N1344H	0.71	0.06	0.02	0.00	0.00	0.00	0.00	0.00	0.00
10	562016	0.52	0.14	PF3D7_1014200	male gamete fusion factor HAP2, putative	D445N	0.85	0.29	0.08	0.05	0.11	0.07	0.02	0.02	0.04
10	563297	0.51	0.057			H872Y	0.74	0.09	0.02	0.02	0.00	0.00	0.00	0.00	0.00

Chr	Pos	Mean F_{ST}	SD F_{ST}	Gene ID	Gene Description	Mutation	AF1	WAF	CAF	EAF	SAS	WSEA	ESEA	OCE	SAM
10	571802	0.56	0.044	PF3D7_1014300	SPRY domain-containing protein, putative	H2000Y	0.86	0.15	0.08	0.09	0.01	0.01	0.02	0.00	0.00
10	578813	0.52	0.047	PF3D7_1014500	conserved Plasmodium protein, unknown function	I745M	0.74	0.07	0.01	0.02	0.01	0.00	0.00	0.00	0.00
10	582046	0.51	0.046	PF3D7_1014600	transcriptional coactivator ADA2	D2349Y	0.72	0.07	0.00	0.01	0.01	0.00	0.00	0.00	0.00
10	653563	0.50	0.02	PF3D7_1016300	glycophorin binding protein	R2Q	0.71	0.04	0.04	0.01	0.00	0.00	0.00	0.00	0.00
10	1038679	0.63	0.016	PF3D7_1024800	exported protein 3	Q1332L	0.88	0.10	0.09	0.07	0.00	0.00	0.01	0.00	0.25
10	1285388	0.51	0.044	PF3D7_1031900	conserved Plasmodium protein, unknown function	F417L	1.00	0.37	0.29	0.30	0.20	0.05	0.01	0.06	0.05
10	1325994	0.66	0.16	PF3D7_1033100	S-adenosylmethionine decarboxylase/ornithine decarboxylase	N815Y	0.98	0.35	0.11	0.09	0.03	0.00	0.00	0.00	0.00
10	1325996	0.66	0.16			E814G	0.98	0.35	0.11	0.09	0.03	0.00	0.00	0.00	0.00
**10**	**1371865**	0.88	0.012	**PF3D7_1034500**	**armadillo repeat protein, putative**	**T336I**	0.98	0.03	0.03	0.05	0.05	0.04	0.00	0.00	0.00
10	1373309	0.55	0.02			I817M	0.97	0.27	0.25	0.23	0.25	0.18	0.36	0.05	0.04
10	1391865	0.51	0.025	**PF3D7_1035100**	**probable protein, unknown function**	S141G	0.02	0.69	0.70	0.74	0.73	0.86	0.85	0.72	0.12
10	1391943	0.59	0.014			N167D	0.02	0.76	0.78	0.79	0.84	0.91	0.93	0.88	1.00
10	1391973	0.58	0.022			N177Y	0.02	0.75	0.78	0.78	0.84	0.91	0.93	0.86	1.00
**10**	**1392014**	0.76	0.034			**H190Q**	0.03	0.88	0.93	0.90	0.90	0.98	0.94	0.90	1.00
10	1392155	0.55	0.017			N237K	0.78	0.04	0.05	0.07	0.04	0.07	0.08	0.35	0.83
10	1399580	0.52	0.069	**PF3D7_1035300**	**glutamate-rich protein GLURP**	D129G	0.00	0.68	0.75	0.61	0.15	0.09	0.09	0.06	0.01
**10**	**1399594**	0.95	0.0088			**S134T**	1.00	0.02	0.02	0.03	0.04	0.03	0.04	0.01	0.89
**10**	**1399634**	0.92	0.01			**V147G**	1.00	0.04	0.03	0.04	0.04	0.03	0.04	0.06	0.89
**10**	**1399636**	0.91	0.024			**Q148E**	1.00	0.04	0.03	0.06	0.04	0.03	0.04	0.11	0.89
**10**	**1399656**	0.95	0.0073			**L154F**	1.00	0.02	0.02	0.03	0.04	0.03	0.04	0.01	0.89
**10**	**1399681**	0.94	0.0058			**S163P**	1.00	0.02	0.03	0.03	0.04	0.03	0.04	0.01	0.89
10	1404580	0.66	0.045	PF3D7_1035400	merozoite surface protein 3	A129V	0.94	0.11	0.11	0.17	0.23	0.28	0.31	0.21	0.10
10	1404591	0.57	0.017			V133F	0.99	0.24	0.25	0.27	0.41	0.44	0.45	0.52	0.10
**10**	**1413597**	0.79	0.0011	**PF3D7_1035700**	**duffy binding-like merozoite surface protein**	**G133D**	0.88	0.00	0.00	0.00	0.00	0.00	0.00	0.00	0.00
**10**	**1413618**	0.84	0.018			**N140S**	0.93	0.00	0.02	0.02	0.00	0.00	0.00	0.00	0.00
10	1413659	0.62	0.04			K154Q	0.96	0.21	0.19	0.15	0.08	0.15	0.09	0.11	0.73
10	1413669	0.54	0.033			L157.	0.91	0.21	0.18	0.15	0.07	0.15	0.08	0.11	0.72
10	1413686	0.73	0.05			N163D	0.91	0.07	0.08	0.02	0.02	0.06	0.01	0.02	0.72
**10**	**1414634**	0.92	0.013			**L479I**	0.97	0.00	0.01	0.02	0.00	0.00	0.00	0.00	0.00
10	1415066	0.58	0.04			K623E	0.97	0.18	0.22	0.26	0.50	0.53	0.47	0.18	0.13
**10**	**1421410**	0.87	0.038	**PF3D7_1035800**	**probable protein, unknown function**	**G293D**	0.97	0.01	0.04	0.06	0.30	0.28	0.26	0.62	0.86
**10**	**1456571**	0.94	0.0051	**PF3D7_1036900**	**conserved Plasmodium protein, unknown function**	**S479I**	0.97	0.00	0.00	0.01	0.00	0.00	0.00	0.00	0.00
**10**	**1468777**	0.80	0.009	**PF3D7_1037000**	**DNA polymerase zeta catalytic subunit, putative**	**N1968S**	0.90	0.01	0.01	0.02	0.00	0.00	0.00	0.00	0.00
**10**	**1483355**	0.92	0.0065	**PF3D7_1037400**		**E667Q**	0.98	0.01	0.02	0.02	0.00	0.00	0.00	0.00	0.00

Chr	Pos	Mean F_{ST}	SD F_{ST}	Gene ID	Gene Description	Mutation	AF1	WAF	CAF	EAF	SAS	WSEA	ESEA	OCE	SAM
10	1485068	0.55	0.052		**conserved Plasmodium protein, unknown function**	S96T	0.87	0.11	0.18	0.10	0.03	0.00	0.00	0.22	0.20
**10**	**1503043**	0.90	0.025	**PF3D7_1037900**	**conserved Plasmodium protein, unknown function**	**N154S**	0.97	0.02	0.04	0.01	0.00	0.00	0.00	0.00	0.07
10	1519152	0.61	0.0034	PF3D7_1038400	gametocyte-specific protein	R44S	0.76	0.00	0.00	0.00	0.00	0.00	0.00	0.00	0.00
**10**	**1548519**	0.89	0.027	**PF3D7_1038500**	**Plasmodium exported protein, unknown function**	**F354C**	0.98	0.05	0.02	0.03	0.11	0.07	0.13	0.32	0.09
10	1552485	0.55	0.077	PF3D7_1038600	Plasmodium exported protein, unknown function	Q190L	0.00	0.79	0.70	0.63	0.32	0.09	0.07	0.80	0.35
10	1552843	0.53	0.0029			F71I	0.70	0.00	0.00	0.00	0.00	0.00	0.00	0.00	0.00
**10**	**1570402**	0.78	0.012	**PF3D7_1039000**	**serine/threonine protein kinase, FIKK family**	**E515A**	0.89	0.02	0.00	0.02	0.00	0.00	0.06	0.00	0.01
11	120959	0.56	0.021	PF3D7_1102500	Plasmodium exported protein (PHISTb)	E110Q	0.95	0.22	0.20	0.24	0.56	0.60	0.93	0.78	0.01
11	123946	0.51	0.0034	PF3D7_1102600	gametocyte exported protein 14	L230I	0.75	0.05	0.05	0.04	0.03	0.00	0.00	0.00	0.00
11	123955	0.51	0.0012			L227F	0.74	0.05	0.05	0.05	0.03	0.00	0.00	0.00	0.00
11	137245	0.52	0.029	PF3D7_1102900	Plasmodium exported protein (hyp11), unknown function	D151Y	0.77	0.04	0.05	0.08	0.00	0.00	0.00	0.00	0.00
11	1484887	0.60	0.027	PF3D7_1137900	conserved Plasmodium protein, unknown function	H505D	0.87	0.07	0.10	0.11	0.01	0.00	0.00	0.01	0.02
11	1640343	0.59	0.079	PF3D7_1140900	conserved Plasmodium protein, unknown function	G865E	0.96	0.27	0.21	0.13	0.03	0.03	0.00	0.00	0.02
11	1642013	0.61	0.062			Y364F	0.96	0.22	0.22	0.13	0.01	0.02	0.00	0.00	0.24
11	1664948	0.57	0.042	PF3D7_1141400	phosphatidylinositol N-acetylglucosaminyltransferase subunit H, putative	D254H	0.85	0.13	0.10	0.07	0.02	0.00	0.00	0.00	0.02
11	1684025	0.56	0.023	PF3D7_1142100	conserved Plasmodium protein, unknown function	N2020K	0.83	0.10	0.09	0.06	0.02	0.01	0.00	0.00	0.00
11	1862558	0.53	0.0037	PF3D7_1147000	sporozoite asparagine-rich protein	D1600E	0.70	0.01	0.00	0.01	0.00	0.00	0.00	0.00	0.00
11	1863226	0.53	0.0033			E1378K	0.70	0.01	0.01	0.01	0.03	0.00	0.00	0.00	0.00
11	1941726	0.55	0.0052	PF3D7_1148800	Plasmodium exported protein (hyp11), unknown function	T86I	0.79	0.06	0.06	0.06	0.53	0.70	0.34	0.88	0.10
11	1956584	0.63	0.0062	**PF3D7_1149000**	**antigen 332, DBL-like protein**	G2047E	0.79	0.01	0.02	0.01	0.00	0.00	0.00	0.00	0.00
11	1957091	0.63	0.003			P2216Q	0.78	0.00	0.01	0.01	0.00	0.01	0.00	0.00	0.00
11	1967527	0.57	0.063			E5695K	0.97	0.21	0.30	0.19	0.02	0.02	0.04	0.01	0.20
**11**	**1967597**	0.94	0.0019			**V5718E**	0.97	0.00	0.00	0.00	0.00	0.00	0.00	0.00	0.00
**11**	**1967951**	0.86	0.0019			**S5836L**	0.92	0.00	0.00	0.00	0.00	0.00	0.00	0.00	0.00
**11**	**1976808**	0.78	0.0019	**PF3D7_1149200**	**ring-infected erythrocyte surface antigen**	**P93A**	0.88	0.00	0.00	0.00	0.00	0.00	0.00	0.00	0.00
11	1977393	0.59	0			Q288E	0.74	0.00	0.00	0.00	0.00	0.00	0.00	0.00	0.00
**11**	**1979012**	0.84	0.06			**K827N**	0.03	0.90	0.98	0.95	0.92	0.99	1.00	0.98	1.00
11	1979127	0.74	0.024			I866L	0.86	0.03	0.00	0.00	0.01	0.00	0.00	0.00	0.00

Chr	Pos	Mean F_{ST}	SD F_{ST}	Gene ID	Gene Description	Mutation	AF1	WAF	CAF	EAF	SAS	WSEA	ESEA	OCE	SAM
11	1979200	0.52	0.058			A890V	0.97	0.26	0.23	0.34	0.74	0.86	0.97	0.98	0.02
**11**	**2001089**	**0.83**	**0.03**			**Y8N**	**0.96**	**0.07**	**0.05**	**0.03**	**0.06**	**0.04**	**0.01**	**0.11**	**0.00**
11	2002527	0.66	0.016			D453E	0.06	0.88	0.85	0.87	0.87	0.77	0.92	0.93	0.71
11	2002901	0.67	0.022			T578I	0.06	0.89	0.86	0.87	0.92	0.89	0.96	0.93	1.00
11	2002933	0.58	0.018	**PF3D7_1149600**	**DnaJ protein, putative**	V589M	0.06	0.83	0.80	0.81	0.84	0.84	0.93	0.88	1.00
11	2002960	0.67	0.026			H598Y	0.06	0.90	0.87	0.86	0.90	0.88	0.95	0.94	1.00
11	2003003	0.67	0.027			A612E	0.06	0.90	0.87	0.86	0.85	0.84	0.91	0.70	1.00
11	2003228	0.62	0.031			A687E	0.06	0.87	0.85	0.82	0.83	0.67	0.90	0.94	1.00
12	73579	0.54	0.003	PF3D7_1200900	Plasmodium exported protein (PHISTc), unknown function	R268.	0.70	0.00	0.00	0.00	0.00	0.00	0.00	0.00	0.00
12	2118264	0.63	0.061	PF3D7_1252100	rhoptry neck protein 3	N1004K	1.00	0.24	0.17	0.28	0.48	0.94	0.69	0.95	0.60
13	83595	0.64	0.0013	PF3D7_1301400	Plasmodium exported protein (hyp12), unknown function	N241K	0.79	0.01	0.00	0.01	0.00	0.00	0.00	0.00	0.00
13	92904	0.61	0.011	PF3D7_1301600	erythrocyte binding antigen-140	H150R	0.77	0.01	0.00	0.01	0.02	0.00	0.00	0.00	0.00
13	127110	0.67	0.057	PF3D7_1302300	Plasmodium exported protein, unknown function	T11I	0.96	0.17	0.09	0.17	0.08	0.05	0.01	0.39	0.93
**13**	**145204**	**0.81**	**0.01**	**PF3D7_1302700**	**ATP-dependent RNA helicase DHR1, putative**	**D11N**	**0.94**	**0.04**	**0.03**	**0.03**	**0.00**	**0.00**	**0.00**	**0.00**	**0.00**
13	593658	0.73	0.03	PF3D7_1313800	conserved Plasmodium membrane protein, unknown function	H1793L	0.91	0.08	0.04	0.05	0.00	0.00	0.00	0.00	0.00
13	612670	0.53	0.023	PF3D7_1314200	telomerase reverse transcriptase	Q447K	0.77	0.05	0.07	0.04	0.00	0.00	0.00	0.00	0.00
13	921466	0.51	0.012	PF3D7_1322100	variant-silencing SET protein	V166I	0.85	0.13	0.12	0.14	0.01	0.00	0.00	0.00	0.00
13	1419303	0.59	0.066	PF3D7_1335100	merozoite surface protein 7	N280T	0.89	0.17	0.07	0.13	0.22	0.15	0.12	0.19	0.56
13	2360342	0.59	0.012	PF3D7_1359400	CUGBP Elav-like family member 1	A286V	0.81	0.05	0.06	0.04	0.02	0.00	0.00	0.00	0.00
13	2515018	0.51	0.032	PF3D7_1362700	conserved Plasmodium protein, unknown function	N1555S	0.79	0.10	0.08	0.06	0.03	0.00	0.00	0.00	0.00
13	2668749	0.50	0.064	PF3D7_1366800	phosphatidylserine synthase, putative	A298V	0.98	0.34	0.22	0.32	0.03	0.01	0.00	0.00	0.11
13	2787976	0.56	0.00071	PF3D7_1370300	membrane associated histidine-rich protein 1	A2E	0.72	0.00	0.00	0.00	0.00	0.00	0.00	0.00	0.00
14	809757	0.70	0.011	PF3D7_1419400	conserved Plasmodium membrane protein, unknown function	S538F	0.87	0.04	0.05	0.03	0.00	0.00	0.00	0.00	0.00
14	810165	0.69	0.014			S402N	0.87	0.04	0.05	0.03	0.00	0.00	0.00	0.00	0.00
14	823710	0.61	0.017	PF3D7_1419800	glutathione reductase	K72N	0.83	0.07	0.05	0.04	0.01	0.00	0.00	0.00	0.00
14	834826	0.62	0.013	PF3D7_1420100	conserved Plasmodium protein, unknown function	S620N	0.85	0.07	0.07	0.05	0.00	0.00	0.00	0.00	0.02
14	844557	0.56	0.024	PF3D7_1420300	Hsp70-escort protein 1	V283L	0.83	0.10	0.06	0.09	0.02	0.00	0.00	0.00	0.00
14	2185212	0.54	0.12			S1198Y	0.85	0.24	0.04	0.09	0.63	0.04	0.00	0.00	0.03
14	2185417	0.57	0.095	PF3D7_1453200	conserved Plasmodium protein, unknown function	N1230I	0.87	0.21	0.06	0.09	0.06	0.28	0.64	0.09	0.02
14	2185421	0.53	0.15			M1231I	0.89	0.35	0.09	0.11	0.85	0.89	0.96	0.79	0.14

Chr	Pos	Mean F_{ST}	SD F_{ST}	Gene ID	Gene Description	Mutation	AF1	WAF	CAF	EAF	SAS	WSEA	ESEA	OCE	SAM
14	2186277	0.53	0.07			K1378N	0.81	0.15	0.04	0.09	0.76	0.94	0.87	0.80	0.01
14	2612645	0.57	0.065	PF3D7_1464500	conserved Plasmodium membrane protein, unknown function	E1785G	0.83	0.14	0.05	0.04	0.02	0.00	0.00	0.00	0.22
14	2638640	0.59	0.066	PF3D7_1465100	conserved oligomeric Golgi complex subunit 6, putative	I359L	0.86	0.15	0.07	0.06	0.05	0.01	0.05	0.00	0.00
14	2639315	0.53	0.064			Y584H	0.85	0.19	0.10	0.09	0.02	0.01	0.04	0.00	0.19
**14**	**2714888**	0.75	0.025	**PF3D7_1466400**	**AP2 domain transcription factor AP2-EXP**	**L800I**	0.89	0.05	0.02	0.02	0.00	0.00	0.00	0.00	0.00
14	2948419	0.74	0.038	PF3D7_1472200	histone deacetylase, putative	N920Y	0.91	0.08	0.05	0.03	0.02	0.01	0.05	0.00	0.00
14	3046529	0.51	0.0025	**PF3D7_1474400**	**conserved Plasmodium protein, unknown function**	T499A	0.70	0.01	0.01	0.02	0.02	0.00	0.00	0.00	0.00
**14**	**3054932**	0.88	0.024			**I2902N**	0.97	0.05	0.03	0.02	0.01	0.00	0.01	0.00	0.03
**14**	**3082419**	0.86	0.028	**PF3D7_1475200**	**conserved protein, unknown function**	**E224K**	0.96	0.05	0.01	0.02	0.00	0.00	0.00	0.00	0.03
14	3088662	0.75	0.025	**PF3D7_1475400**	**cysteine repeat modular protein 4**	A5374S	0.91	0.04	0.04	0.07	0.00	0.00	0.00	0.00	0.00
14	3088877	0.76	0.014			I5302K	0.91	0.04	0.04	0.05	0.00	0.00	0.00	0.00	0.00
**14**	**3097632**	0.75	0.0069			**Q2384E**	0.90	0.03	0.04	0.03	0.00	0.00	0.01	0.00	0.00
**14**	**3183699**	0.90	0.0025	**PF3D7_1477400**	**Plasmodium exported protein (PHIST), unknown function**	**H87Y**	0.95	0.00	0.01	0.00	0.00	0.00	0.00	0.00	0.00
14	3193593	0.58	0.05	PF3D7_1477600	surface-associated interspersed protein 14.1 (SURFIN 14.1)	K103N	0.97	0.19	0.21	0.28	0.28	0.17	0.22	0.28	0.30
14	3193676	0.51	0.018			R131K	0.98	0.30	0.27	0.30	0.23	0.25	0.50	0.26	0.26
14	3193843	0.66	0.026			L187I	0.95	0.12	0.16	0.16	0.10	0.12	0.05	0.06	0.00
14	3194436	0.50	0.032			N325D	0.00	0.66	0.64	0.70	0.56	0.72	0.81	0.75	0.97

Supplementary Table 3 – Correlation between AF1 characteristic loci.

A symmetrical matrix showing the r^2 measure of linkage disequilibrium between selected SNPs at seven AF1 characteristic loci; each locus is represented by the single SNP with the highest mean r^2 with respect to the remaining five loci. The SNP coordinates of each SNP are shown in the form "*chr*:*position*". The remaining columns show: The ID and description of the gene containing the SNP; whether the SNP is nonsynonymous or synonymous, and the amino acid change if any; the mean F_{ST} between AF1 and each of WAF, CAF and EAF populations; and the allele frequency of the AF1 allele in each of the populations, followed by the frequency in other populations present in the Pf7 dataset (SAS=South Asia; WSEA=Western Greater Mekong Subregion; ESEA=Eastern Greater Mekong Subregion; OCE=Oceania; SAM=South America).

SNP	02:814192	09:1205151	10:1413597	11:1984241	12:73579	13:2787976	14:3183699	Variant				F_{ST}	Allele Frequencies								
								Gene ID	Gene Description	N/S	Name		AF1	WAF	CAF	EAF	SAS	WSEA	ESEA	OCE	SAM
02:814192	-	0.51	0.59	0.49	0.42	0.53	0.47	PF3D7_0220300	Plasmodium exported protein, unknown function	N	P92A	0.56	0.72	0	0	0.01	0	0	0	0	0
09:1205151	0.51	-	0.58	0.66	0.43	0.58	0.50	PF3D7_0930300	merozoite surface protein 1	N	N1114Y	0.91	0.96	0.01	0	0	0	0	0	0	0.1
10:1413597	0.59	0.58	-	0.73	0.51	0.70	0.56	PF3D7_1035700	duffy binding-like merozoite surface protein	N	G133D	0.79	0.88	0	0	0	0	0	0	0	0
11:1984241	0.49	0.66	0.73	-	0.52	0.78	0.66	PF3D7_1149300	serine/threonine protein kinase, FIKK family	S	559K	0.73	0.85	0	0	0	0	0	0	0	0
12:73579	0.42	0.43	0.51	0.52	-	0.40	0.50	PF3D7_1200900	Plasmodium exported protein (PHISTc), unknown function	S	R268	0.54	0.7	0	0	0	0	0	0	0	0
13:2787976	0.53	0.58	.70	0.78	0.40	-	0.50	PF3D7_1370300	membrane associated histidine-rich protein 1	N	A2E	0.56	0.72	0	0	0	0	0	0	0	0
14:3183699	0.47	0.50	0.56	0.66	0.50	0.50	-	PF3D7_1477400	Plasmodium exported protein (PHIST), unknown function	N	H87Y	0.9	0.95	0.01	0	0	0	0	0	0	0

Supplementary Table 4 – High-IBD genomic regions in the AF1 group

Each row in this table represents one genome region where ≥50% of AF1 sample pairs are in IBD. From left to right, the columns show: the chromosome number, start position, end position and size of the region; the highest proportion of AF1 sample pairs in IBD in this region, and the position where it occurs; the highest mean F_{ST} (vs the WAF, CAF and EAF populations) in this region, the position where it occurs, the ID and description of the gene, whether the change is synonymous or not, the mutation caused, and the frequency of the non-reference allele in the AF1 population. The regions are sorted in descending order of mean F_{ST}. All positions are with respect to the 3D7 v3 reference genome.

Region				Max IBD pairs		Maximum F_{ST} Position						
Chr	Start	End	Size	% pairs	Pos	F_{ST}	Pos	ID	Description	N/S	Mut	AF1Freq
10	1335368	1571788	236421	95.7%	1442027	0.94	1456571	PF3D7_1036900	conserved Plasmodium protein, unknown function	N	S479I	0.97
9	1171854	1226411	54558	85.7%	1202410	0.93	1205284	PF3D7_0930300	merozoite surface protein 1	N	V1158E	0.97
14	3183284	3207686	24403	87.5%	3183284	0.90	3183699	PF3D7_1477400	Plasmodium exported protein (PHIST), unknown function	N	H87Y	0.95
9	1378245	1455635	77391	95.4%	1427653	0.90	1427697	PF3D7_0936000	ring-exported protein 2	N	S77.	0.98
14	3044149	3114489	70341	85.0%	3080648	0.88	3054932	PF3D7_1474400	conserved Plasmodium protein, unknown function	N	I2902N	0.97
4	92597	124976	32380	88.4%	110070	0.85	103881	PF3D7_0401800	Plasmodium exported protein (PHISTb), unknown function	N	K515R	0.93
2	299208	342734	43527	60.2%	306058	0.85	306406	PF3D7_0207600	serine repeat antigen 5	N	K159E	0.93
11	1934420	2003312	68893	84.1%	1985877	0.83	2001089	PF3D7_1149600	DnaJ protein, putative	N	Y8N	0.96
13	83595	167851	84257	67.2%	121740	0.81	145204	PF3D7_1302700	ATP-dependent RNA helicase DHR1, putative	N	D11N	0.94
1	558230	569858	11629	87.2%	558230	0.80	563776	PF3D7_0114700	PIR protein	N	A300V	0.93
2	769929	814641	44713	79.6%	769929	0.79	784067	PF3D7_0219700	gametocyte exported protein 20	N	Y182H	0.89
9	777971	807125	29155	73.0%	781952	0.75	781952	PF3D7_0919000	nucleosome assembly protein	N	I76V	0.87
14	803248	836620	33373	75.7%	803248	0.70	809757	PF3D7_1419400	conserved Plasmodium membrane protein, unknown function	N	S538F	0.87
8	1311766	1345474	33709	80.5%	1311766	0.68	1311901	PF3D7_0830800	surface-associated interspersed protein 8.2 (SURFIN 8.2)	N	P422R	0.95
2	109623	153841	44219	56.2%	109623	0.65	153549	PF3D7_0203100	protein kinase, putative	N	E1145K	0.80
3	841714	866401	24688	52.6%	848289	0.63	865666	PF3D7_0320700	signal peptidase complex subunit 2	N	M78L	0.89
14	2585522	2672686	87165	67.5%	2635842	0.62	2668129	PF3D7_1465800	dynein beta chain, putative	S	1710C	0.81
7	705498	718468	12971	60.0%	708260	0.59	714223	PF3D7_0716200	PDCD2 domain-containing protein, putative	S	585G	0.77
6	847157	888840	41684	67.4%	847157	0.59	851783	PF3D7_0620400	merozoite surface protein 10	N	K391N	0.89
5	55996	128576	72581	60.8%	64857	0.55	93127	PF3D7_0501800	CAF-1 p150 homolog	S	589T	0.87
11	1856542	1866942	10401	54.3%	1856542	0.53	1862558	PF3D7_1147000	sporozoite asparagine-rich protein	N	D1600E	0.70
11	123841	138994	15154	58.6%	137023	0.51	123946	PF3D7_1102600	gametocyte exported protein 14	N	L230I	0.75
10	653023	682454	29432	55.6%	669207	0.50	653563	PF3D7_1016300	glycophorin binding protein	N	R2Q	0.71

Supplementary Table 5 – Functional Enriched Gene Ontology (GO) Terms in genes carrying SNPs highly differentiated in AF1.

This table shows Gene Ontology (GO) terms whose proportions were significantly higher (p≤0.05) among annotations of genes carrying highly differentiated AF1 characteristic SNPs (Supplementary Table 2) than in the remaining genes. The columns show: the GO term category, identifier and description, and the Benjamini-Hochberg-corrected p-value of a one-sided Fisher's Exact Test for enrichment (see Supplementary Text for details).

Category	GO Term	Description	*p*
**Biological Process**	GO:0044409	symbiont entry into host	<0·0001
	GO:0035891	exit from host cell	0.00096
	GO:0050776	regulation of immune response	0.0011
	GO:0002377	immunoglobulin production	0.0019
	GO:0045454	cell redox homeostasis	0.0078
	GO:0007004	telomere maintenance via telomerase	0.020
	GO:0006465	signal peptide processing	0.026
	GO:0010468	regulation of gene expression	0.026
	GO:0030522	intracellular receptor signaling pathway	0.026
	GO:0032958	inositol phosphate biosynthetic process	0.026
**Molecular Function**	GO:0005515	protein binding	<0·0001
	GO:0046812	host cell surface binding	<0·0001
	GO:0008201	heparin binding	<0·0001
	GO:0046789	host cell surface receptor binding	<0·0001
	GO:0044877	protein-containing complex binding	0.0026
	GO:0008234	cysteine-type peptidase activity	0.027
	GO:0001791	IgM binding	0.027
	GO:0004791	thioredoxin-disulfide reductase (NADP) activity	0.027
	GO:0016668	oxidoreductase activity, acting on a sulfur group of donors, NAD(P) as acceptor	0.027
**Cellular Component**	GO:0009986	cell surface	<0·0001
	GO:0030430	host cell cytoplasm	<0·0001
	GO:0043657	host cell	<0·0001
	GO:1903561	extracellular vesicle	<0·0001
	GO:0020009	microneme	<0·0001
	GO:0044538	host cell periphery	<0·0001
	GO:0020036	Maurer's cleft	0.00048
	GO:0044228	host cell surface	0.00048
	GO:0020003	symbiont-containing vacuole	0.0033
	GO:0020008	rhoptry	0.0033
	GO:0005787	signal peptidase complex	0.0046
	GO:0000123	histone acetyltransferase complex	0.012
	GO:0033644	host cell membrane	0.023

SUPPLEMENTARY FIGURES

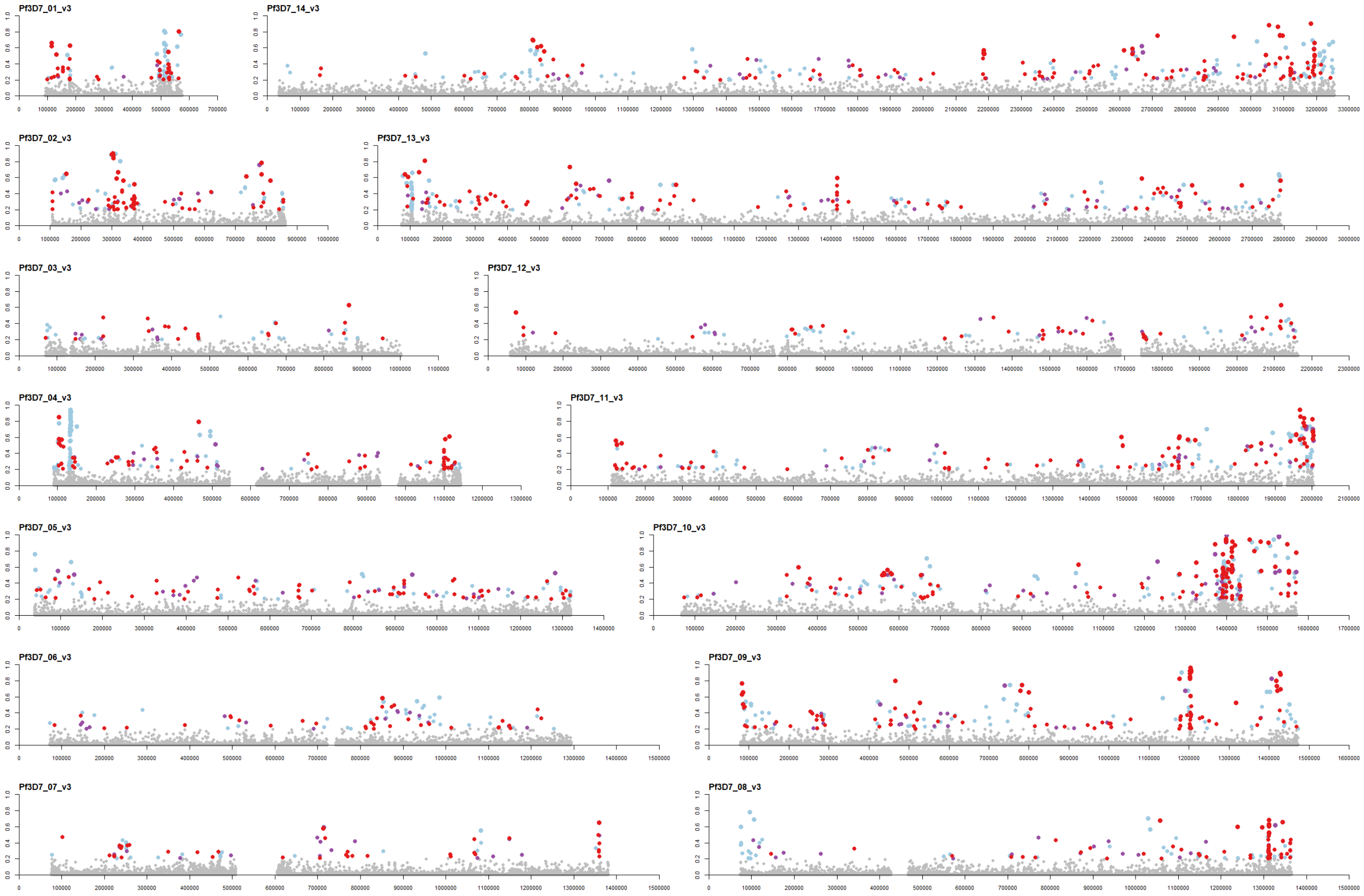

Supplementary Figure 1 – Genome-wide map of F_{ST} between AF1 and other African populations.
These plots (one per chromosome, as labelled in the upper left-hand corner of each plot) show the mean F_{ST} between AF1 and the three African macro-regions (WAF, CAF and EAF) at 743,583 SNPs. At each position, we plotted the F_{ST} value (between 0 and 1). Positions with $F_{ST} \geq 0.2$ are shown by markers coloured according to the type of SNP: light blue for non-coding, purple for synonymous coding, and red for non-synonymous coding variants; SNPs with $F_{ST} < 0.2$ are shown by gray markers.

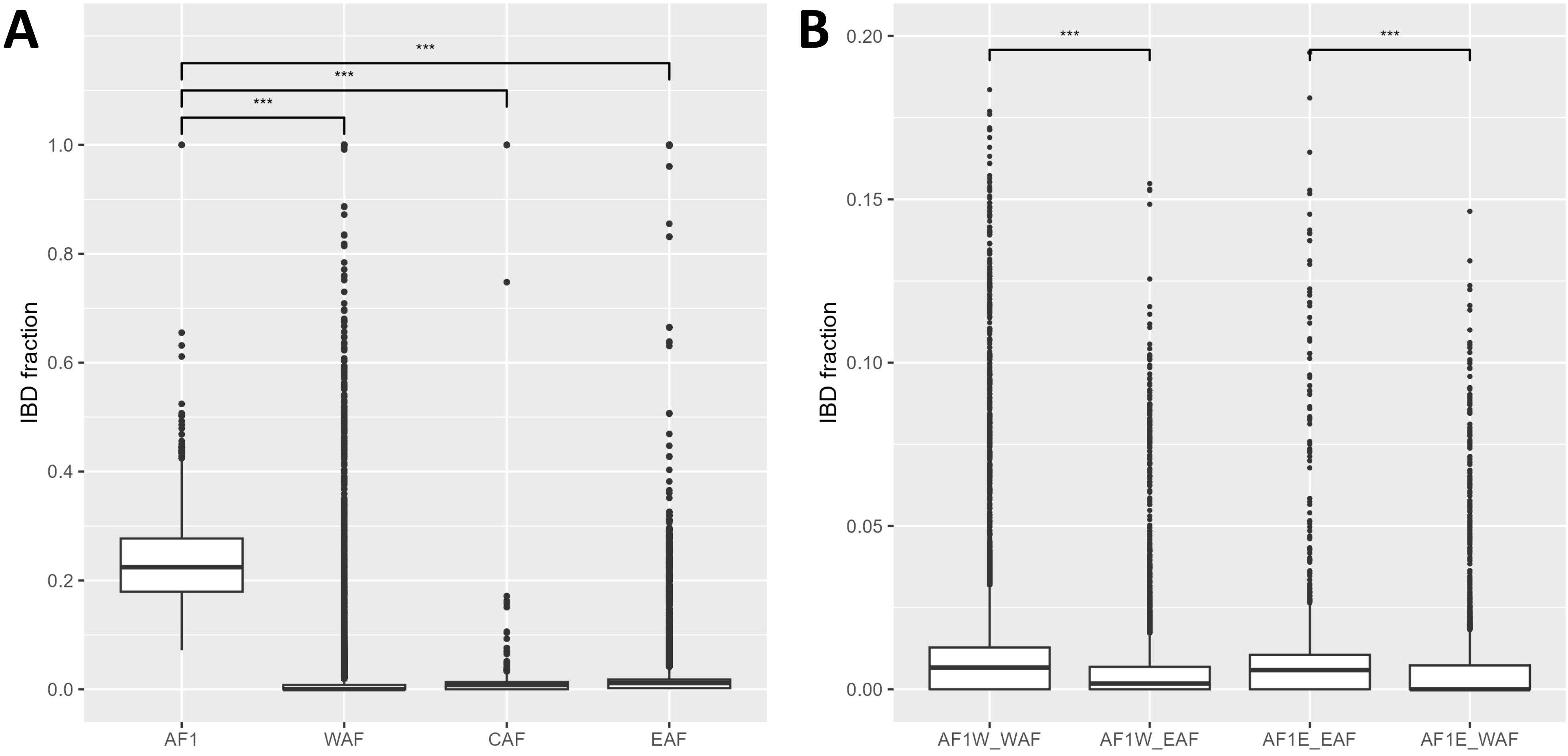

Supplementary Figure 2 – Pairwise IBD fraction levels within and between African populations.
(A) Boxplot showing the distribution of IBD genome fractions between all pairs of parasites in each of four populations: AF1, West Africa (WAF), Central Africa (CAF) and East Africa (EAF). Levels within AF1 are significantly higher than within the rest of the populations ($p<0.001$). (B) Boxplot showing the distribution of IBD genome fractions between populations. The first two columns show IBD fractions for all pairings of West African AF1 members (AF1W) with West African (column 1) and East African (column 2) non-AF1 parasites (WAF and EAF respectively). The remaining two columns show the IBD fractions for all pairings of East African AF1 members (AF1E) with East African (column 3) and West African (column 4) non-AF1 parasites. Although IBD levels between populations are low, they are significantly higher between AF1 members and non-AF1 parasites from the same regions than those from a different region.

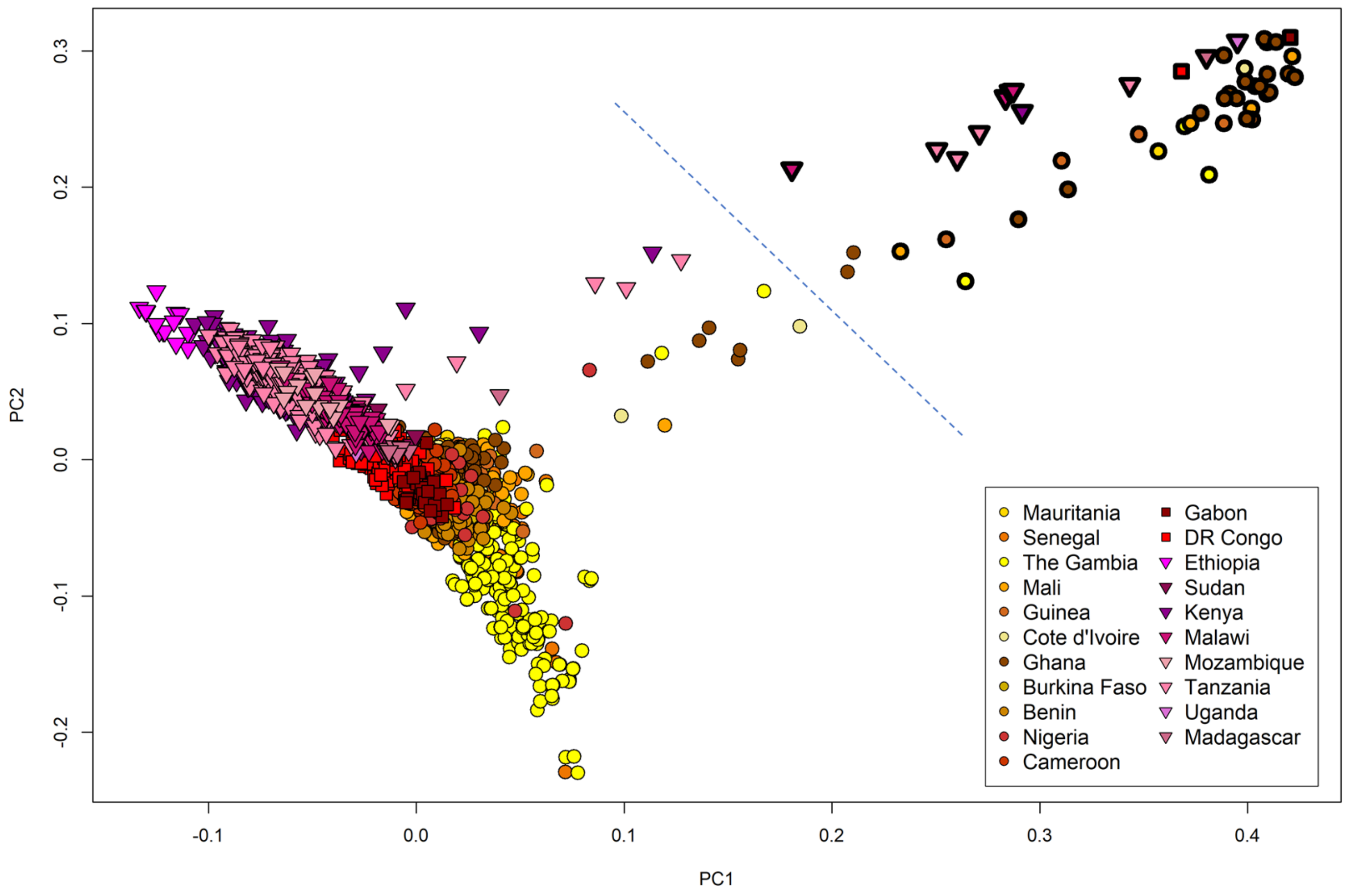

Supplementary Figure 3 – PCoA plot based on an IBD distance measure.
This figure shows the first two components of a PCoA derived from an NxN pairwise genetic distance matrix (N=3,783), where pairwise distance was calculated as d=(1-f_{IBD}) where f_{IBD} is the fraction of the genome that is predicted to be IBD in a given pair. Samples are coloured by country of origin, and AF1 parasites are shown with a thicker border. A blue dotted line shows a cut-off between AF1 and non-AF1 parasites; AF1 samples clearly form a highly compact outlier group.

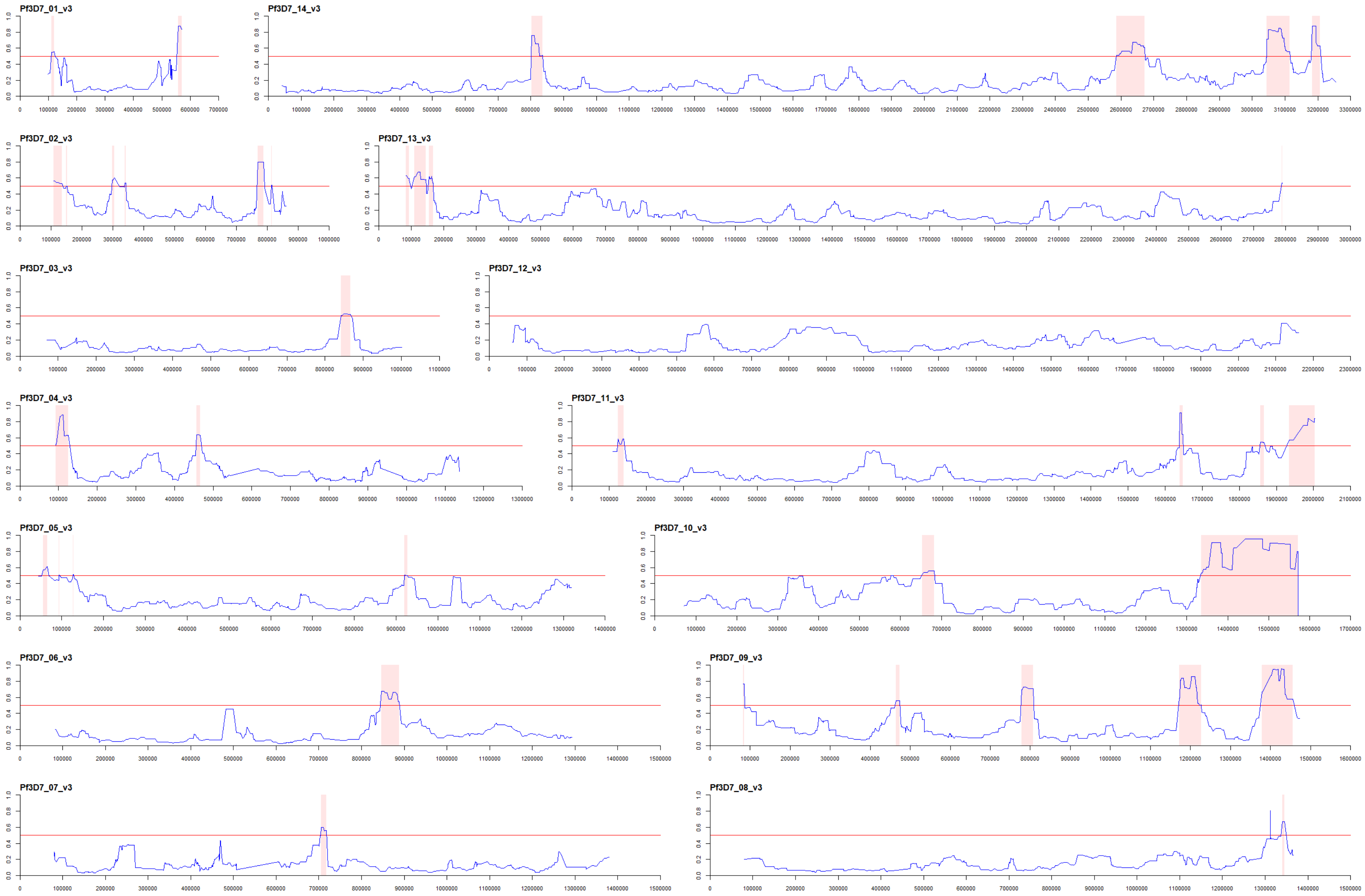

Supplementary Figure 4 – Genome-wide map of pairwise IBD within AF1.

The 14 plots (one per chromosome, as labelled in the upper left-hand corner of each plot) show the proportion of AF1 sample pairs that are predicted to be identical by descent at 43,469 SNPs (blue line). Region with ≥50% IBD sample pairs (red threshold line) are highlighted by a pink background.

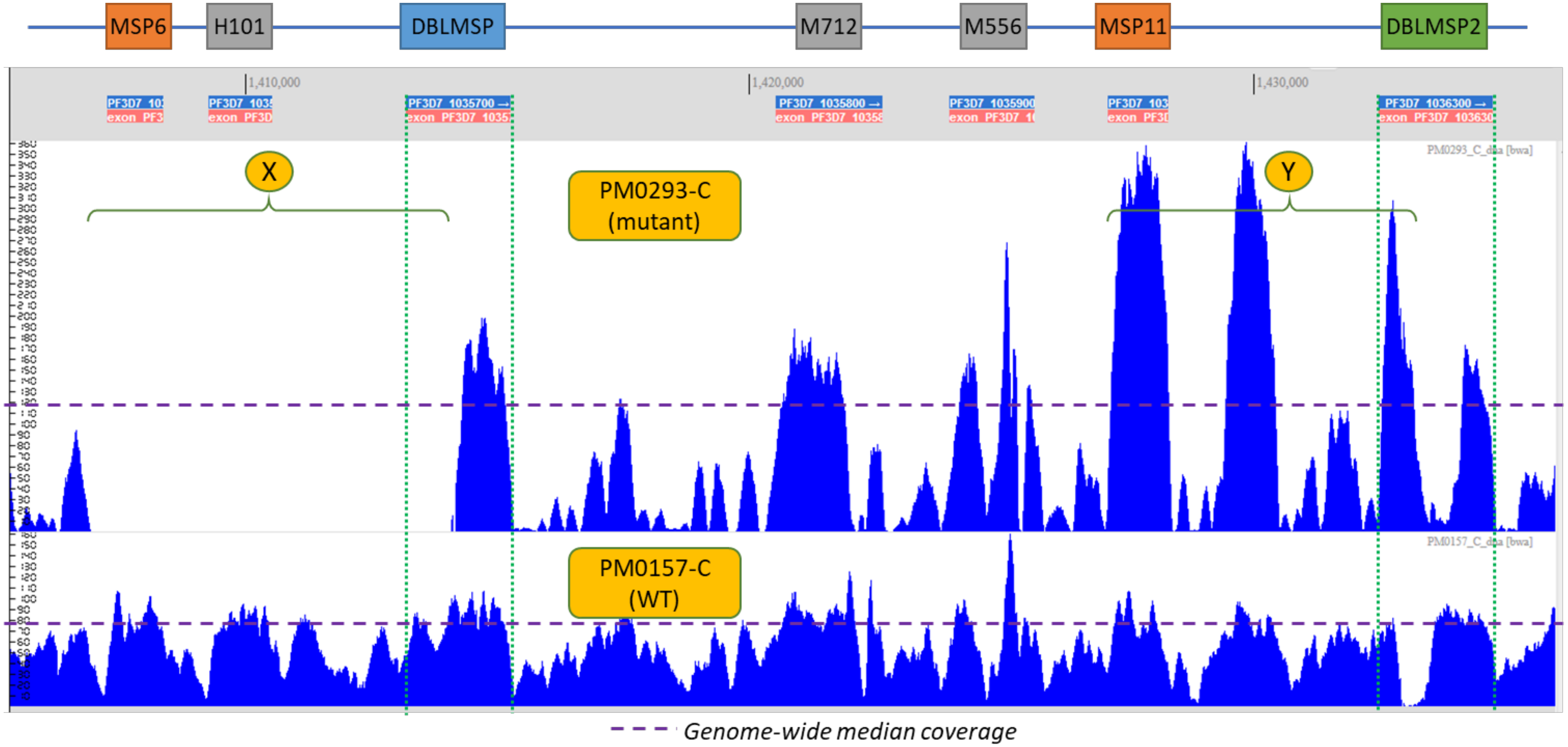

Supplementary Figure 5 – Coverage of the Chromosome 10 locus in an AF1 sample.
The above figure shows two pileup plots for the largest AF1 characteristic locus on chromosome 10 (visualized using the LookSeq genome browser[10]). The height of the pileup indicates the read coverage (as shown on the y-axis). The upper plot shows the pileup for reads from the PM0293-C AF1 member from Mali, while the lower plot shows the pileup for PM0157-C, a non-AF1 parasite from Mali. The genome coordinates (relative to the Pf3D7 reference genome) and the extent of the genes covered by this plot are shown above the pileup plots, topped by coloured boxes showing the genes' names. Purple dashed lines show the genome-wide median read coverage for the two samples. Two regions are demarcated: region "X" shows no coverage over genes MSP6 and H101, and over the 5' end of DBLMSP, suggesting a large deletion; and region "Y" shows high coverage over MSP11 and the 5' end of DBLMSP2.

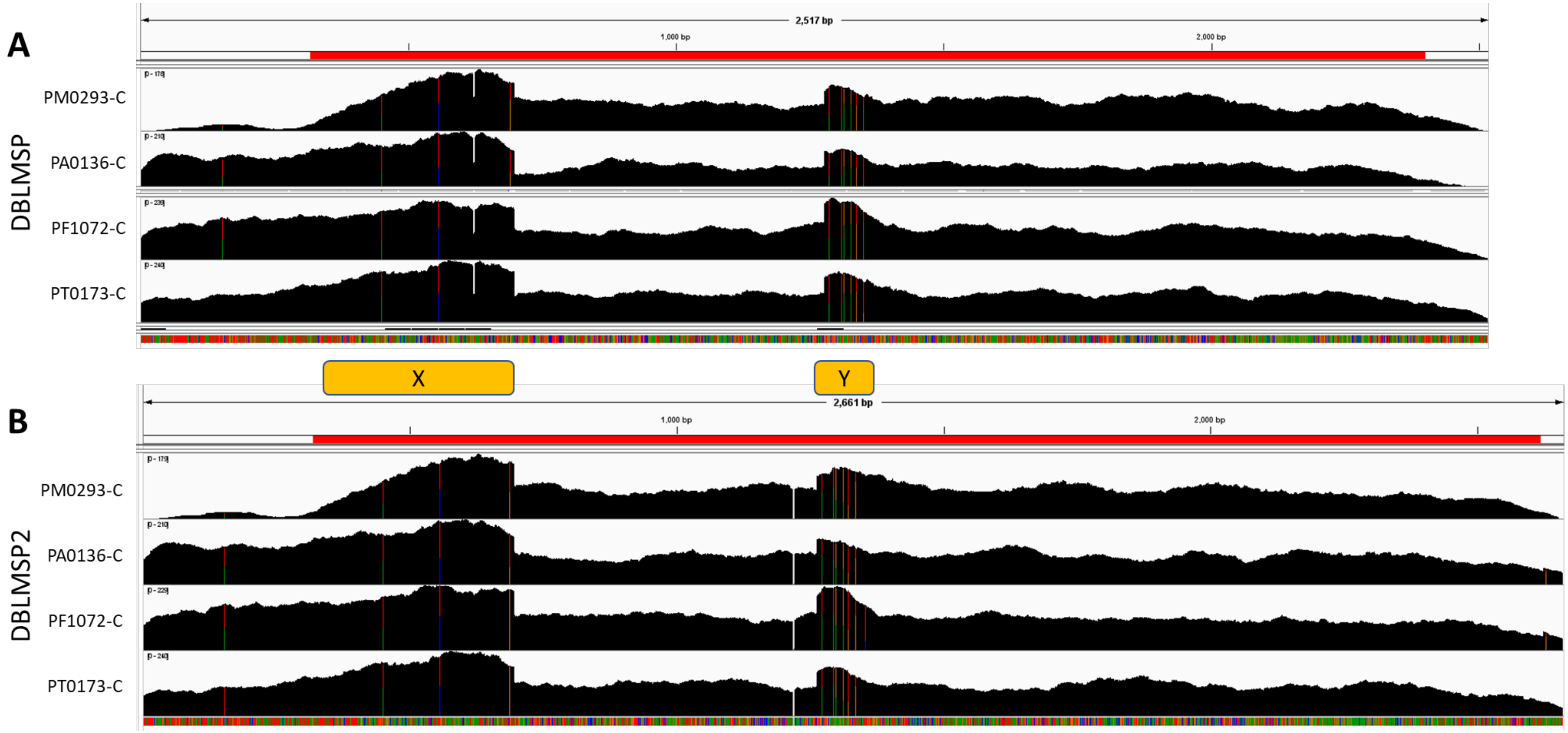

Supplementary Figure 6 – Coverage profiles of AF1 sequencing read alignments on predicted *de novo* assembly reads.
The two panels show plots of coverage in alignments of sequencing reads for four AF1 samples (PM0293-C, PA0136-C, PF1072-C and PT173-C) using as references the *de novo* assembled sequences of DBLMSP (panel A) and DBLMSP2 (panel B) from AF1 sample PM0293-C (see Supplementary Text). The red stripe in the upper section of each panel indicates the coding sequence of the gene used as reference. The alignments were conducted separately to avoid alignment competition. For both genes, all four samples show even coverage, without sizeable coverage gaps, over most of the coding sequence. This contrasts with lack of coverage in the 5' regions of DBLMSP when aligned against Pf3D7 (Supplementary Figure 7), consistent with a correct assembly of the AF1 sequences. At the 5' end of each alignment (denoted by "X"), there is an approximate doubling in coverage, consistent with the two genes having near-identical sequences in that region, such that reads sequenced from both genes map to the region. A similar effect is observed in the region containing the breakpoint sequence ("Y"). The visualizations were created using the Integrative Genomics Viewer (IGV)[4].

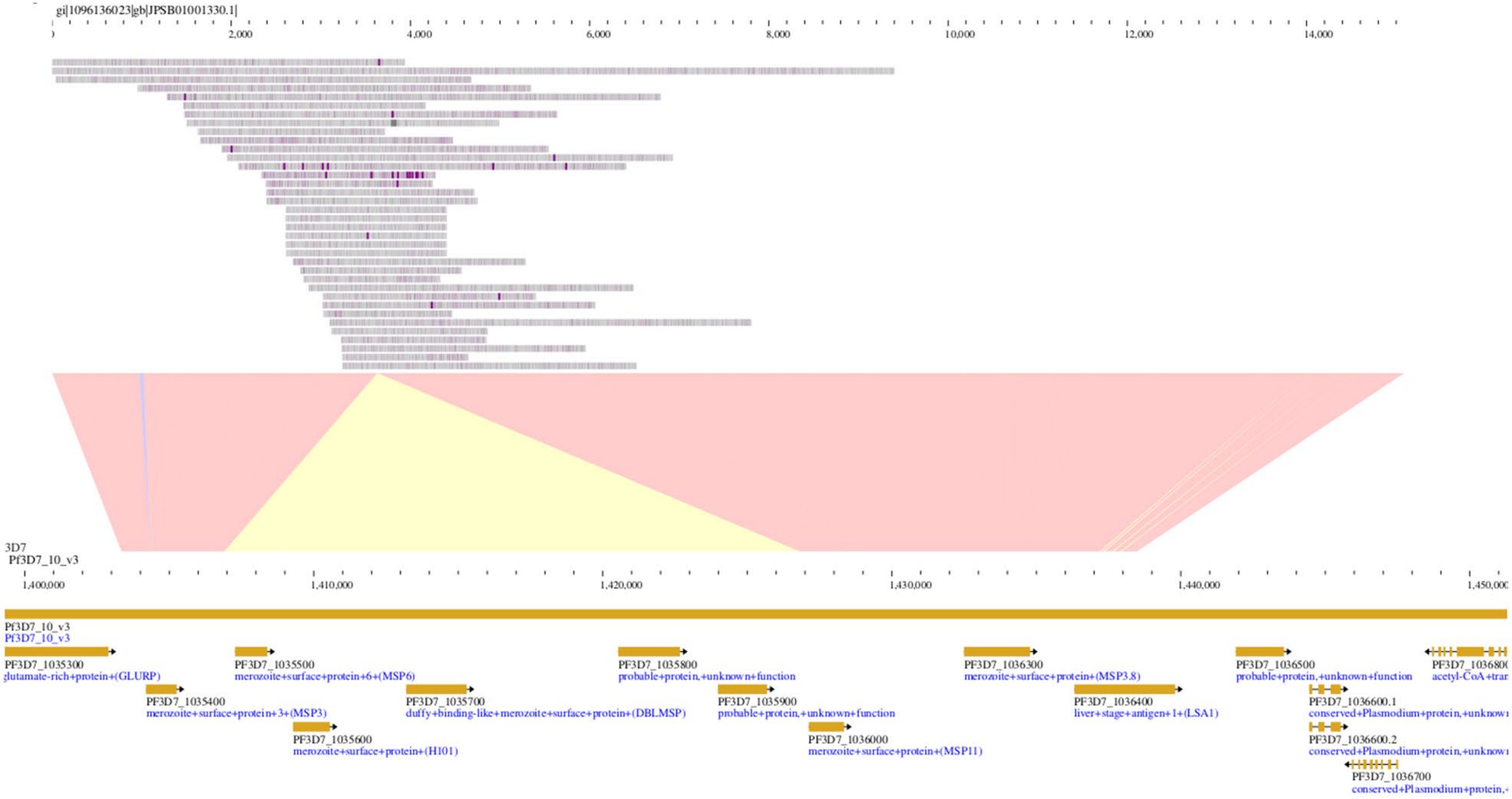

Supplementary Figure 7 – Long-read confirmation of a large deletion at the AF1 Chromosome 10 locus.
This synteny diagram shows the mapping of contig JPSB01001330.1 (~15kbp length) built from PacBio long reads (pileup detail shown in top panel) from AF1 sample 318.1 (see Supplementary Text) onto Chromosome 10 of the Pf 3D7 reference genome (gene positions shown in bottom panel). Pink shapes indicate correspondence between contig and reference, while the yellow shape map a large (~19kbp) deletion comprising 5 genes (see Supplementary Text for details). The span of the mapped long reads confirms the predicted presence of the deletion.

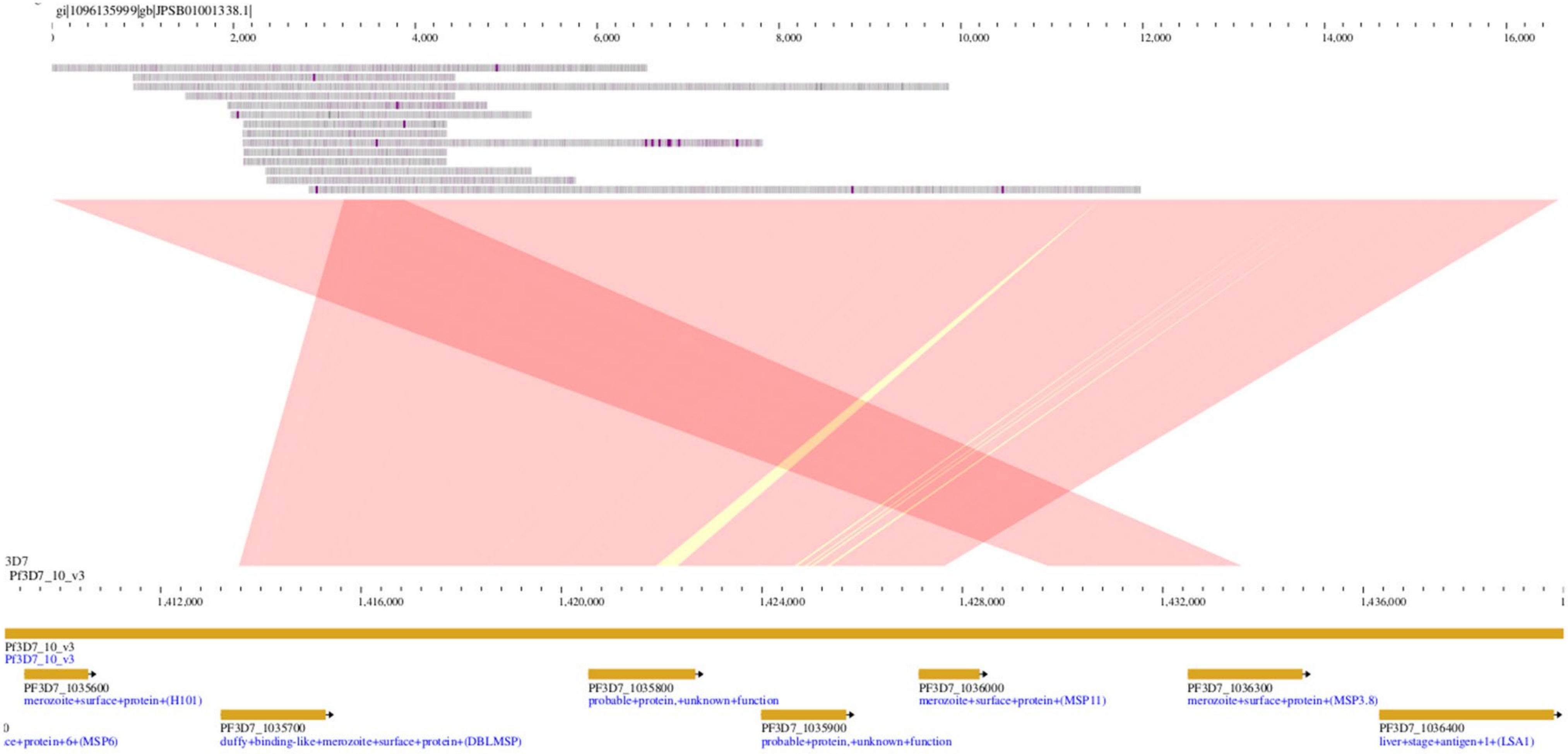

Supplementary Figure 8 – Long-read confirmation of the DBLMSP/DBLMSP2 gene conversion at the AF1 Chromosome 10 locus.
This synteny diagram shows the mapping of contig JPSB01001338.1 (~15kbp length) built from PacBio long reads (pileup detail shown in top panel) from AF1 sample 318.1 (see Supplementary Text) onto Chromosome 10 of the Pf 3D7 reference genome (gene positions shown in bottom panel). Pink shapes show a recombination event between gene DBLMSP2 (PF3D7_1036300) and DBLMSP (PF3D7_1035700) occurring in a region of overlap, as predicted by *de novo* alignments (Figure 4). In addition, two of the genes that appeared to be deleted in another contig (Supplementary Figure 9) are present in this contig, indicating they were involved in a genomic rearrangement. The span of the mapped long reads confirms the predicted overlap of DBLMSP2 and DBLMSP in the region where they recombine.

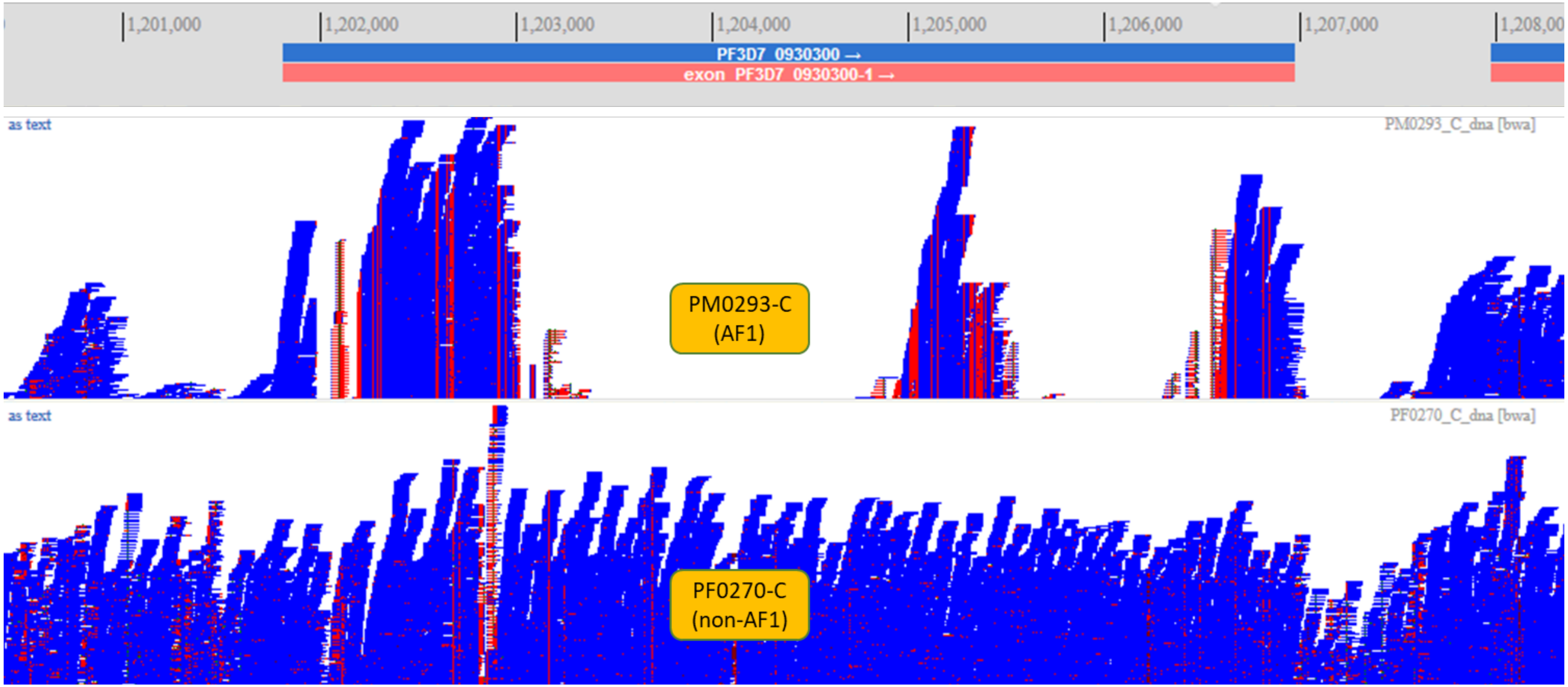

Supplementary Figure 9 – Alignment of AF1 sequencing reads in the MSP1 gene.
The above figure shows two pileup plots (visualized using the LookSeq genome browser[10]) in which the height of the pileup indicates the read coverage. The upper plot shows the pileup for reads from the PM0293-C AF1 member from Mali, while the lower plot shows the pileup for PF0270-C, a non-AF1 parasite from Ghana. The genome coordinates (relative to the Pf3D7 reference genome) and the extent of the MSP1 gene (Pf3D7_0930300) are shown above the pileup plots. The large gaps in the AF1 pileup denote regions of the gene (blocks) where the PM0290-C sequence is highly differentiated with respect to the Pf3D7 reference, to the extent that sequencing reads cannot be mapped. This does not occur in the PF0270-C genome, whose sequence is similar to that of Pf3D7.

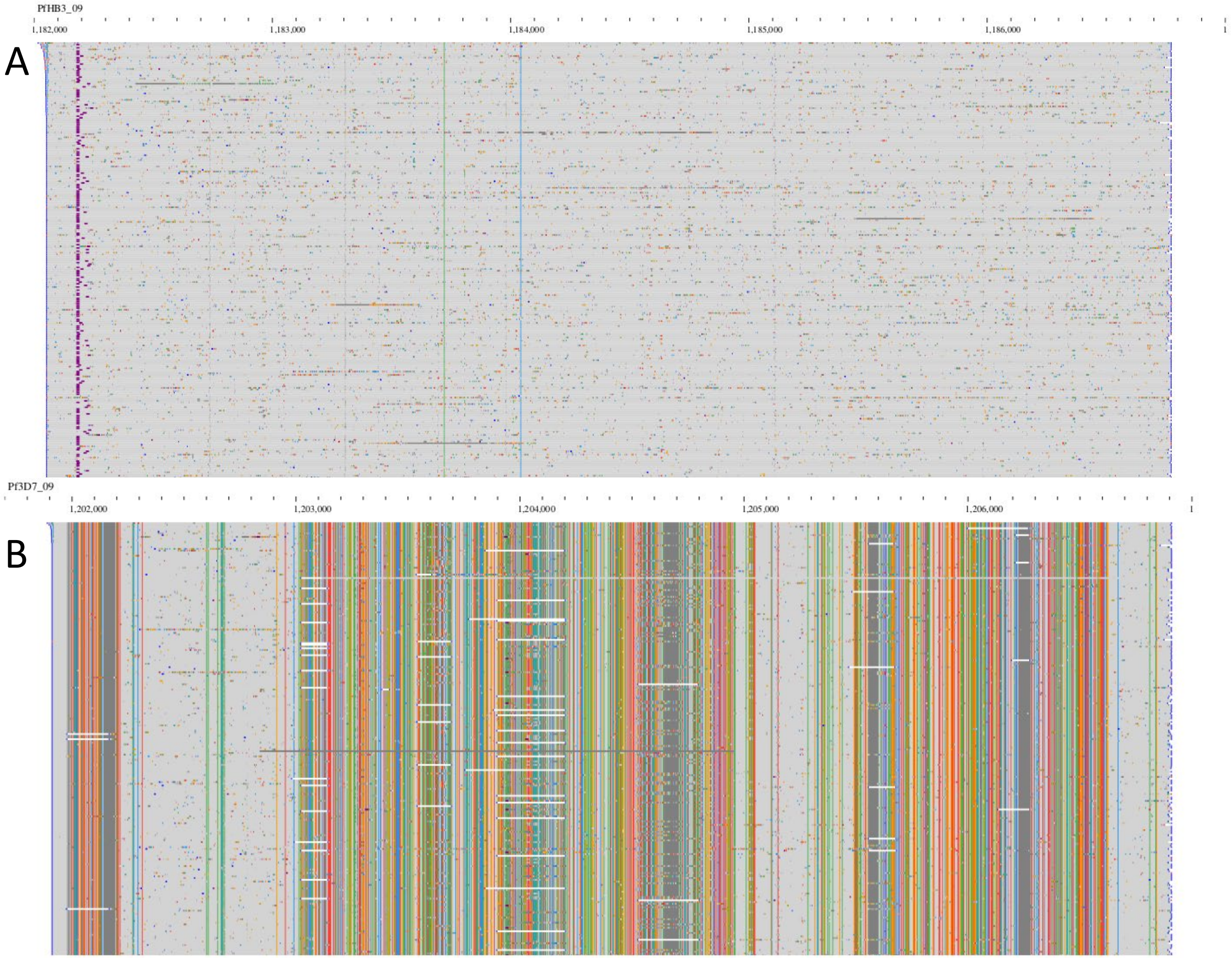

Supplementary Figure 10 – Alignment of AF1 long sequencing reads against two different MSP1 gene references.

In the above figure, the two panels show alignments of long sequencing reads from amplicon sequencing of the MSP1 gene in an AF1 sample (SPT15471 from Tanzania, see Supplementary Text) against the MSP1 genes from two reference strains: HB3 (Panel A) and 3D7 (B). Each horizontal line shows a single amplicon read (length ~4800bp); gray positions indicate agreement with the reference sequence, while coloured positions indicate a non-reference allele. The AF1 reads are essentially identical to the HB3 sequence except for two SNPs, and a short indel in a highly variable region; in contrast, most of the 3D7 MSP1 sequence is markedly different from that in the AF1 reads, explaining why AF1 short reads could not map evenly when using the 3D7 reference (Supplementary Figure 11)

REFERENCES FOR SUPPLEMENTARY MATERIALS

1. Oyola SO, Ariani CV, Hamilton WL, et al. Whole genome sequencing of Plasmodium falciparum from dried blood spots using selective whole genome amplification. *Malar J* 2016; **15**(1): 597.
2. Hill WG, Robertson A. Linkage disequilibrium in finite populations. *Theoret Appl Genet* 1968; **38**: 226-31.
3. Krzywinski M, Schein J, Birol I, et al. Circos: an information aesthetic for comparative genomics. *Genome Res* 2009; **19**(9): 1639-45.
4. Robinson JT, Thorvaldsdottir H, Winckler W, et al. Integrative genomics viewer. *Nat Biotechnol* 2011; **29**(1): 24-6.
5. Dara A, Drabek EF, Travassos MA, et al. New var reconstruction algorithm exposes high var sequence diversity in a single geographic location in Mali. *Genome Med* 2017; **9**(1): 30.
6. Danecek P, Bonfield JK, Liddle J, et al. Twelve years of SAMtools and BCFtools. *Gigascience* 2021; **10**(2).
7. Li H. Minimap2: pairwise alignment for nucleotide sequences. *Bioinformatics* 2018; **34**(18): 3094-100.
8. Diesh C, Stevens GJ, Xie P, et al. JBrowse 2: a modular genome browser with views of synteny and structural variation. *Genome Biol* 2023; **24**(1): 74.
9. Girgis ST, Adika E, Nenyewodey FE, et al. Drug resistance and vaccine target surveillance of Plasmodium falciparum using nanopore sequencing in Ghana. *Nat Microbiol* 2023; **8**(12): 2365-77.
10. Manske HM, Kwiatkowski DP. LookSeq: a browser-based viewer for deep sequencing data. *Genome Res* 2009; **19**(11): 2125-32.
